# Progressive suppression of regulated cell death defines terminal macrophage states in liver cirrhosis

**DOI:** 10.1080/07853890.2026.2690717

**Published:** 2026-06-25

**Authors:** Kepu Zheng, Leiyang Dai, Yanghui Wen, Haitao Jiang, Yang Gao, Feng Ren, Yu Tang, Xiang Huang, Jianghua Ran, Yunjie Chen

**Affiliations:** a Department of Hepato-Biliary-Pancreatic Surgery, Ningbo No.2 Hospital, Ningbo, China; b Department of Clinical Laboratory, the First Affiliated Hospital of Kunming Medical University, Kunming, China; c Department of Hepato-Biliary-Pancreatic Surgery, The Affiliated Calmette Hospital of Kunming Medical University, The First People’s Hospital of Kunming, Kunming, China; d Department of Genetics, Zunyi Medical University, Zunyi, China; e Department of Medical Experiment, Ningbo No.2 Hospital, Ningbo, China

**Keywords:** Liver cirrhosis, macrophages, single-cell RNA sequencing, programmed cell death, metabolic reprogramming, diagnostic biomarkers

## Abstract

**Background:**

Macrophages are central regulators of liver immunity and fibrosis; Recent single-cell studies have characterized macrophage heterogeneity in cirrhosis; however, how regulated cell death programs shape their functional transitions across disease progression in human liver remains poorly defined.

**Methods:**

We integrated scRNA-seq data from 224,529 cells across 31 adult liver samples to define macrophage states and trajectories. Functional activities of regulated cell death pathways were assessed, and key programs were validated by dual immunofluorescence in human liver tissues.

**Results:**

Four macrophage subsets with disease-biased distributions were identified. Cirrhotic macrophages exhibited progressive suppression of autophagy, necroptosis, apoptosis, and immunogenic cell death along differentiation, most prominently in terminal states, accompanied by transcriptional exhaustion and reduced immune regulator expression. A disease-associated transitional subpopulation displayed hybrid metabolic–immune features and yielded a six-gene diagnostic signature (LDHB, CD37, S100A10, RNASE1, CXCR4 and CORO1A) validated in bulk RNA-seq data (AUC = 0.936). Spatial analyses confirmed attenuation of autophagy and necroptosis and enrichment of CXCR4- and S100A10-positive macrophages in fibrotic niches.

**Conclusion:**

Human cirrhosis is characterized by progressive repression of regulated cell death programs in liver macrophages, providing mechanistic insight into disease progression and identifying macrophage-associated molecular signatures that may aid in patient stratification and serve as potential targets for future therapeutic intervention.

## Introduction

Liver cirrhosis represents the end stage of chronic liver disease and remains a leading cause of morbidity and mortality worldwide [1]. Marked by progressive fibrosis, vascular remodeling, regenerative nodules, and immune disruption, it leads to portal hypertension, hepatic encephalopathy, and hepatocellular carcinoma [2]. Globally, cirrhosis is responsible for more than one million deaths annually and represents a substantial contributor to years of life lost worldwide, highlighting its considerable public health burden [1]. Although antiviral treatments, such as tenofovir, have shown potential to regress fibrosis and even cirrhosis in some patients, there are still no broadly effective therapies capable of consistently reversing advanced cirrhosis [3]. In addition to nucleos(t)ide analogues such as entecavir, other antiviral strategies and investigational antifibrotic therapies, including interferon-based regimens and metabolism-targeted approaches, have been explored [4–7]; however, their efficacy in reversing or slowing the progression of advanced cirrhosis remains limited. Emerging evidence suggests that dysregulated immune responses and remodeling of the hepatic microenvironment play central roles in disease progression [8].

Among hepatic immune components, macrophages, including resident Kupffer cells and recruited monocyte-derived subsets, mediate both fibrogenesis *via* TGF-β–driven stellate cell activation and tissue repair through phagocytosis, cytokine release, and matrix degradation [9,10]. Although the classical M1/M2 paradigm offers a simplified view of macrophage function, human tissue macrophages exhibit far greater transcriptional diversity [11]. Critically, programmed cell death pathways, especially autophagy and necroptosis, regulate macrophage survival and function in chronic liver disease [12]. However, their roles in shaping macrophage subsets during human cirrhosis progression remain underexplored.

Recent single-cell RNA-seq studies have revealed heterogeneous macrophage states, including inflammatory and immunoregulatory phenotypes, underscoring the importance of metabolic programming in macrophage function [13]. Notably, previous landmark single-cell liver atlases have comprehensively mapped hepatic immune heterogeneity [14]; but, these studies primarily focused on cell identity and inflammatory activation states rather than systematically resolving how regulated cell death programs dynamically evolve during macrophage differentiation in cirrhosis. Despite these advances, a comprehensive single-cell analysis of macrophage cell death programs, specifically autophagy and necroptosis, in the context of established human cirrhosis is still lacking. The interplay between death-related pathways and macrophage differentiation trajectories in human diseases is a critical knowledge gap.

Animal and *in vitro* models demonstrate that autophagy governs macrophage activation, phagocytosis, and cytokine secretion, while necroptosis promotes sterile inflammation and fibrosis exacerbation [15,16]; hepatocyte necroptosis also contributes to fibrogenesis [17]. Although macrophages are known to play a central role in liver fibrosis, the specific contribution of macrophage death to cirrhosis pathogenesis remains poorly understood. Furthermore, the spatiotemporal activation of these pathways among macrophage subsets and their contribution to the disease trajectory in human livers remain to be elucidated. In addition, the local hepatic microenvironment, including hypoxia, extracellular matrix stiffness, and mechanical stress, can actively remodel programmed cell death signaling and macrophage fate decisions, further emphasizing the importance of spatial context in cirrhosis progression [18–20].

Single-cell transcriptomic technologies offer unprecedented opportunities to resolve these questions. They enable the reconstruction of cellular differentiation trajectories, quantification of pathway activity at single-cell resolution, and the discovery of rare or disease-specific populations. Although recent human liver atlases have captured immune heterogeneity, they primarily focus on cirrhosis-associated endothelial cells or T cells [21]. A systematic analysis that connects macrophage subtype dynamics with autophagy and necroptosis activity and that validates key findings in independent bulk transcriptomic datasets has not yet been performed.

Here, we integrated single-cell RNA sequencing data from 31 human liver samples, comprising both healthy and cirrhotic tissues across three public datasets to construct a comprehensive atlas of hepatic macrophages in cirrhosis. By applying the functional scoring of cell death pathways, pseudotime inference, and subtype-resolved transcriptomic analyses, we sought to elucidate how programmed cell death shapes macrophage phenotypes during fibrotic progression. We further aimed to identify pathological macrophage states associated with cirrhosis and define the potential roles of autophagy and necroptosis in regulating macrophage fate in the diseased liver.

## Materials and methods

### Data sources and sample overview

This study integrated single-cell RNA sequencing (scRNA-seq) data from 31 adult human liver samples, comprising 17 healthy individuals and 14 patients with liver cirrhosis. Publicly available datasets were retrieved from the GEO and ArrayExpress repositories GSE136103 [22], GSE181483 [23], and E-MTAB-10553 [24]. Among them, GSE181483 was generated using the 10x Genomics platform (3′ v3 chemistry) and sequenced on the Illumina NovaSeq 6000. GSE136103 also utilized the 10x Genomics platform with sequencing on HiSeq 4000; and E-MTAB-10553 provided preprocessed ‘.rds’ files derived from the HiSeq 2500. The detailed metadata and publication references are listed in Supplementary Table 1.

### Data preprocessing and quality control

All three datasets provide author-preprocessed gene expression matrices. These were directly downloaded in either count matrix or ‘.rds’ format from GEO or ArrayExpress for downstream analyses. Based on the original metadata and associated publications, the reference genomes used were GRCh38.p13 for GSE181483, GRCh38 for GSE136103, and Ensembl GRCh38.93 for E-MTAB-10553. All data preprocessing was conducted using Python (v3.10.17) and R (v4.3.2) with primary packages including Scanpy (v1.11.1) [25], scvi-tools (v1.3.0) [26], and Seurat (v5.1.0) [27].

The quality control criteria were tailored for each dataset as follows: GSE181483: cells with >5% mitochondrial content, <500 or >6000 detected genes were removed; GSE136103: cells with >10% mitochondrial content, <300 or >5000 genes were filtered; E-MTAB-10553: cells with >15% mitochondrial content, <200 or >4000 genes were excluded, and genes expressed in <10 cells were removed to minimize background noise. The relatively higher mitochondrial RNA threshold applied to E-MTAB-10553 (15%) was chosen based on the overall quality distribution of this dataset. Specifically, this dataset exhibited a higher baseline mitochondrial read fraction and broader variability in mitochondrial content across immune cell populations, likely reflecting differences in tissue processing and sequencing chemistry. To avoid excessive removal of biologically relevant macrophage subpopulations, a slightly relaxed cutoff was therefore adopted, consistent with previously reported dataset-specific quality control strategies in liver single-cell atlases. Potential doublets were identified using ‘scrublet’ (v0.2.3) [28] and filtered according to dataset-specific thresholds. After quality control, 26,848 cells from GSE181483, 61,501 cells from GSE136103, and 136,180 cells from E-MTAB-10553 were retained, resulting in 224,529 high-quality cells for downstream analysis.

### Integration and batch correction

Data integration and batch effect correction were performed using scvi-tools (v1.3.0). The unified AnnData object was modeled using ‘scvi.model.SCVI.setup_anndata()’ followed by ‘model.train()’ to learn a latent representation while correcting for donor-level batch effects. The resulting latent space was used for dimensionality reduction *via* UMAP (′sc.pl.umap()′), and to evaluate donor mixing and cross-sample consistency. For specific downstream analyses (e.g. macrophage subclustering), separate scVI models were also constructed on cell subsets to enhance the resolution of intragroup structures.

### Clustering and cell-type annotation

Initial clustering was performed using the Leiden algorithm (′tl.leiden()′) in Scanpy, with the resolution parameter set to 0.25. Automated cell-type annotation was conducted using CellTypist (v1.6.3) [29], followed by manual curation of canonical marker genes. Sixteen major cell types were identified, including T cells, B cells, macrophages, NK/NKT cells, dendritic cells, hepatocytes, cholangiocytes, fibroblasts, and endothelial cells. The cluster and annotation metadata were subsequently imported into the Seurat object in R for further analysis.

### Differential abundance analysis

Cell-type abundance per donor was calculated using the ‘dplyr’ package (v1.1.4) in R (v4.3.2). Comparisons of cell-type proportions between healthy and cirrhotic groups were performed using the Wilcoxon rank-sum test at the donor level to identify significantly altered populations.

### Macrophage subclustering and pathway scoring

Macrophages were extracted and reclustered using Seurat’s ‘FindNeighbors()’ and ‘FindClusters()’ functions (resolution = 0.27), followed by UMAP visualization of cluster distribution. Functional scoring was conducted using AUCell (v1.24.0) [30] to compute the AUC scores across the 12 literature-curated cell death pathways (Supplementary Table 2). The active/inactive states were defined using the Global_k1 classification strategy. At the sample level, single-sample gene set enrichment analysis (ssGSEA) was performed using the ‘GSVÀ package (v1.50.1) [31] to quantify the enrichment scores of programmed cell death pathways (e.g. autophagy and necroptosis) in healthy and cirrhotic conditions.

### Trajectory inference and differentiation analysis

Lineage reconstruction of macrophage subsets was performed using Slingshot (v2.10.0) [32]. Differentiation potential was first assessed by calculating CytoTRACE (v0.3.3) [33] scores, with Macrophage-1 (highest CytoTRACE score) selected as the trajectory root. Lineage trajectories were fitted in a low-dimensional UMAP space, and tradeSeq (v1.22.0) [34] was applied to model gene expression dynamics over pseudotime, using generalized additive models (GAMs) with 6 knots, following default recommendations. This allowed for the identification of key regulators associated with differentiation potential and immune reprogramming.

### Pathway enrichment and protein–protein interaction analysis

Differentially expressed genes (DEGs) were identified using ‘Seurat::FindMarkers()’ with Wilcoxon testing (log_2_FC > 0.25, adj. *p* < 0.05). KEGG pathway enrichment analysis was performed using ‘clusterProfiler’ (v4.10.1) [35], with a FDR cutoff of <0.05. Protein–protein interaction (PPI) networks were constructed using the STRING database [36] and visualized in Cytoscape (v3.10.3) [37], with hub genes ranked by degree centrality. Module detection and GO enrichment analysis were conducted using the Metascape platform [38] (https://metascape.org/).

### Validation and biomarker modeling

Marker genes significantly upregulated in Macrophage-4 cluster 3 (identified by ‘Seurat::FindMarkers()′, log_2_FC > 0.25, adj. *p* < 0.05) were validated in an independent bulk liver transcriptomic dataset (GSE89377). This dataset comprises transcriptome profiles from human hepatocellular carcinoma (HCC) tissues and corresponding non-tumorous liver samples. HCC is a malignancy that typically arises in the context of chronic liver disease and represents the terminal stage of a multistep hepatocarcinogenic process driven by long-term inflammatory and fibrotic remodeling. Therefore, this dataset was used to assess whether the macrophage-associated gene signature identified in cirrhosis is also relevant across the chronic liver disease progression spectrum, particularly during malignant transformation. A LASSO regression model was constructed using the ‘glmnet’ package (v4.1-8) [39], and classification performance was evaluated using the ‘pROC’ package (v1.18.5) [40], including AUC, sensitivity, and specificity metrics.

### Immunofluorescence staining and image quantification

Human liver sections were collected from the Department of Hepato-Biliary Surgery at the First People’s Hospital of Kunming from March 2024. Liver tissue samples, including healthy control and cirrhotic specimens, were collected from patients undergoing clinically indicated surgical resection or liver biopsy procedures after obtaining written informed consent. All procedures were approved by the Medical Ethics Committee of the First People’s Hospital of Kunming (Approval No. YLS2024-026).

Collected tissues were immediately fixed in 10% neutral-buffered formalin and embedded in paraffin according to standard histopathological protocols. For immunofluorescence staining, FFPE sections were deparaffinized, rehydrated, and subjected to antigen retrieval when necessary, followed by blocking in serum-containing blocking buffer.

Sections were incubated with the following primary antibodies: CD68 (Proteintech, 25747-1-AP, rabbit, 1:200), LC3B (Proteintech, 14600-1-AP, rabbit, 1:500), p62/SQSTM1 (Proteintech, 18420-1-AP, rabbit, 1:1000), phospho-RIPK3 (abmart, TA3894S, rabbit, 1:200), CXCR4 (Proteintech, 60042-1-Ig, mouse, 1:300), CD37 (Proteintech, 84509-2-RR, rabbit, 1:300), S100A10 (Proteintech, 11250-1-AP, rabbit, 1:400), and COL1A1 (Proteintech, 67288-1-Ig, mouse, 1:400). Appropriate species-matched secondary antibodies conjugated with Cy3 or FITC (Proteintech, SA00009-1, SA00009-2, SA00003-1, SA00003-2; each used at 1:100 dilution) were applied. Nuclear counterstaining was performed using Hoechst (Beyotime, C1011, 1:1000). Slides were mounted using antifade mounting medium.

**Figure 1.**
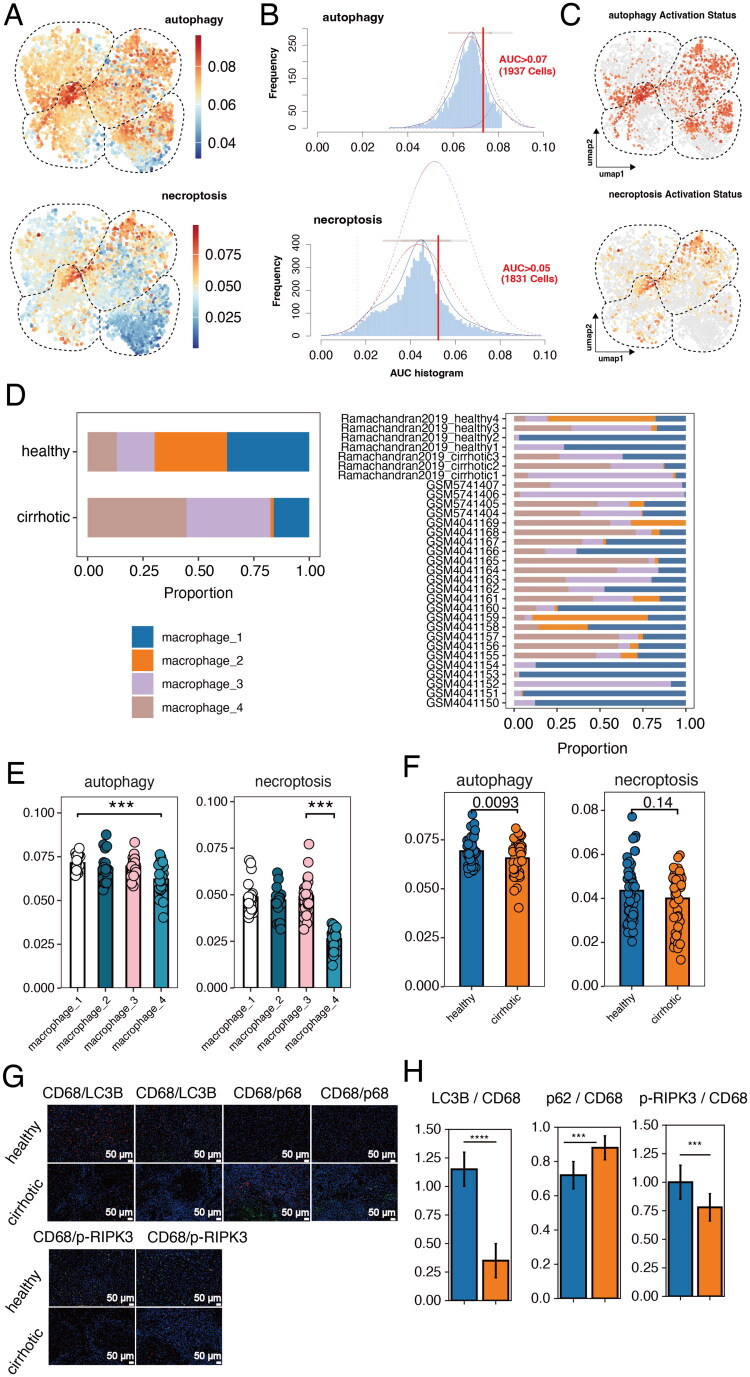
Distribution and cell death state of macrophages in different disease groups. (A) UMAP plots of AUCell scores for autophagy and necroptosis in macrophages, with higher scores shown in red; (B) Threshold values used to define activation of autophagy and necroptosis; (C) UMAP plots showing activation status: red indicates activation, grey indicates no activation; (D) Proportion of four macrophage subclusters in healthy and cirrhotic groups (left), and across 31 samples (right); (E) AUCell score comparison for autophagy and necroptosis across the four macrophage subclusters; (F) AUCell scores of autophagy and necroptosis in healthy vs. cirrhotic patients (p-values shown in the figure). (G) Representative dual immunofluorescence images showing CD68 with LC3B, p62/SQSTM1, and p-RIPK3 (each as a 2-plex staining with DAPI) in healthy and cirrhotic liver tissues. (H) Combined quantification of LC3B/CD68, p62/CD68, and p-RIPK3/CD68 signals.

**Figure 2.**
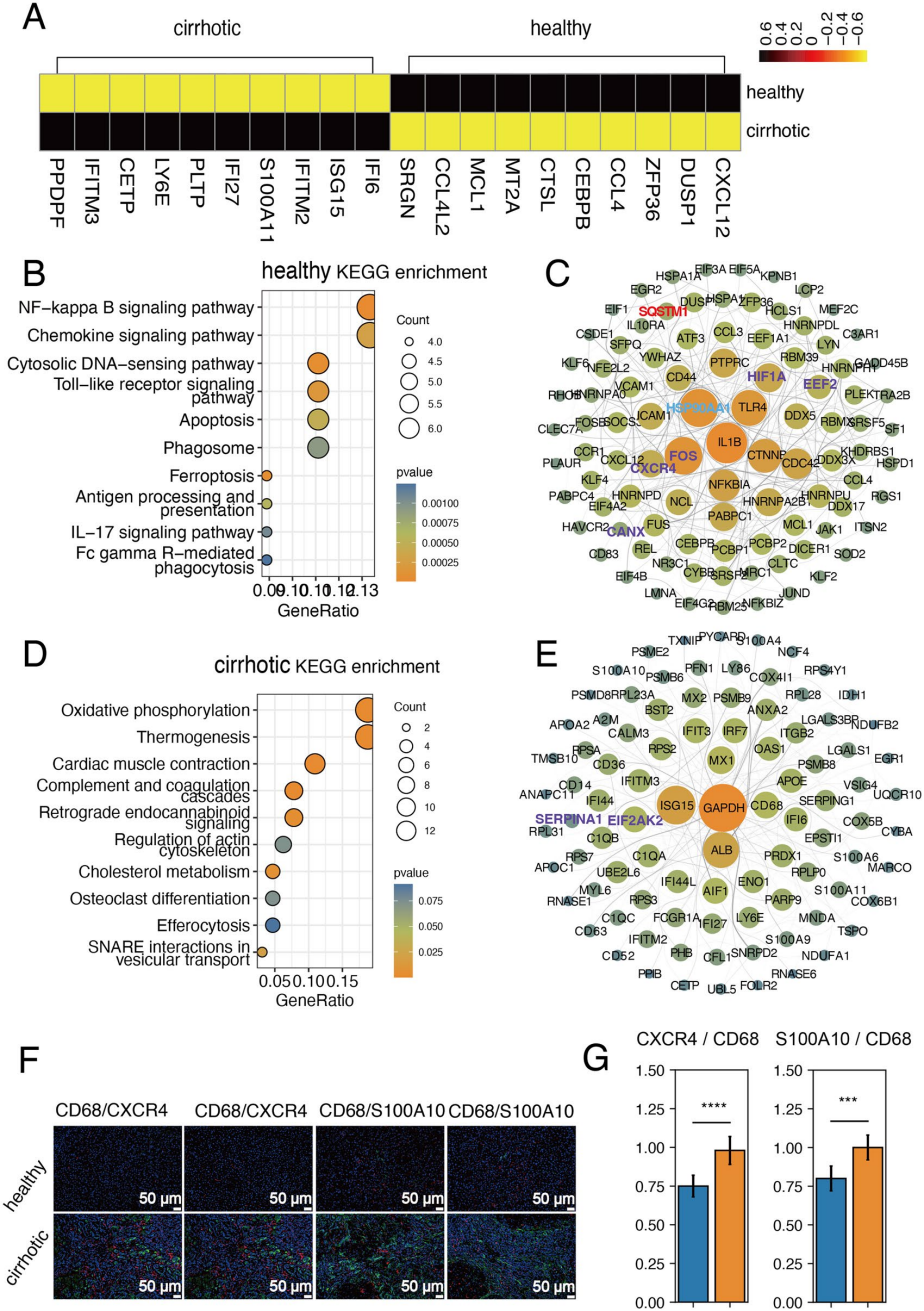
Functional differences and key marker genes in macrophages from healthy vs. cirrhotic samples. (A) Marker gene identification in macrophages comparing healthy and cirrhotic groups; darker color indicates higher expression; (B) Functional enrichment of healthy-associated marker genes; (C) Interaction network of healthy-associated marker genes; purple = autophagy-related, blue = necroptosis-related, red = both; (D) Functional enrichment of cirrhosis-associated marker genes.; (E) Interaction network of cirrhosis-associated marker genes.(F) Representative dual immunofluorescence images showing CD68 with CXCR4 and S100A10 (each as a 2-plex staining with DAPI) in healthy and cirrhotic liver tissues. (G) Combined quantification of CXCR4/CD68 and S100A10/CD68 within CD68-positive regions.

**Figure 3.**
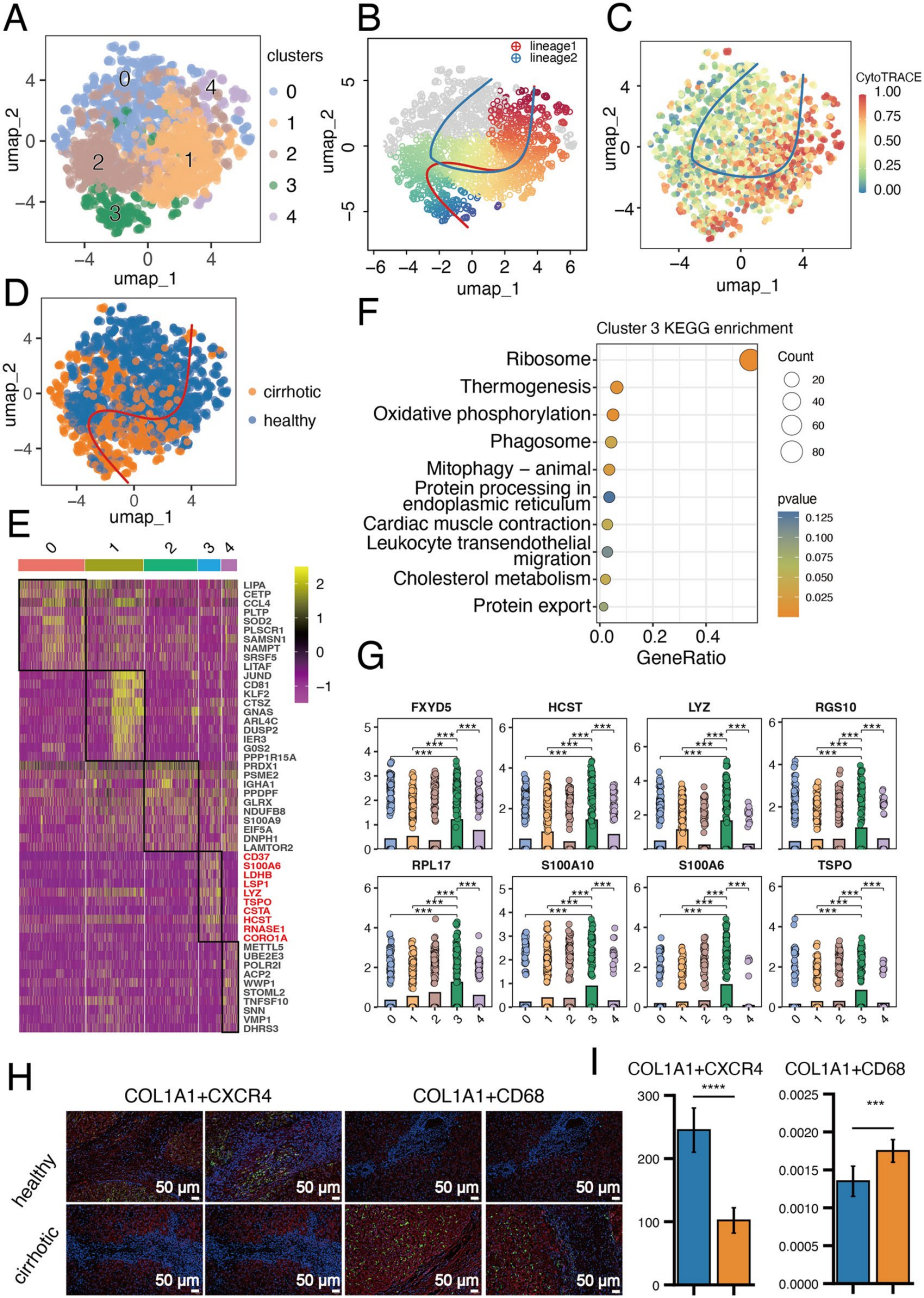
Subclustering, trajectory, and key gene identification for terminally differentiated macrophages. (A) UMAP plot showing subdivision of Macrophage-4 into five clusters; (B) Slingshot trajectory analysis of Macrophage-4; red = start, blue = end; (C) CytoTRACE-colored UMAP plot showing differentiation potential of Macrophage-4 across five clusters; red = less differentiated, green = more mature. Blue line indicates Slingshot lineage 2; (D) UMAP plot showing healthy and cirrhotic sample distributions within Macrophage-4. Red line indicates Slingshot lineage 1; (E) Heatmap showing differentially expressed genes across the five Macrophage-4 clusters; yellow = higher expression; (F) GO enrichment of DEGs in cluster 3; (G) Differential expression of marker genes across the five Macrophage-4 subclusters. (H) Immunofluorescence staining showing CXCR4-positive signal and CD68-positive macrophages relative to COL1A1-positive fibrotic septa (co-staining or serial adjacent sections with matched fields), with DAPI. (I) Quantification of relative distance to fibrotic septa and septa-adjacent macrophage density.

Images were acquired under identical acquisition settings within each staining batch. For Figure 1G–H and Figure 2F–G, fluorescence signals were quantified within CD68-positive regions and expressed as CD68-normalized ratios (LC3B/CD68, p62/CD68, p-RIPK3/CD68, CXCR4/CD68, and S100A10/CD68). For Figure 3H–I, COL1A1 staining was used to delineate fibrotic septa; spatial analysis included the distance of CXCR4-positive cells to the nearest COL1A1-positive septal boundary and the density of CD68-positive macrophages in peri-septal regions. Quantification was performed using Fiji/ImageJ with consistent threshold settings. Statistical analyses are described in the Statistical Analysis section.

### Visualization

The figures were generated using built-in functions of Scanpy and Seurat. Additional customization was performed using ‘ggplot2’ (v3.5.0) and ‘pheatmap’ (v1.0.12) in R. KEGG tree plots were created using WeightedTreemaps (v0.1.2), and PPI networks were visualized in Cytoscape (v3.10.3).

### Statistical analysis

Statistical analyses were performed in R. Unless otherwise specified, non-parametric Wilcoxon rank-sum test was used for comparisons between two groups. P values were adjusted for multiple testing using the Benjamini–Hochberg false discovery rate (FDR) correction where applicable. For single-cell differential gene expression analysis, Wilcoxon rank-sum test implemented in Seurat was used. All reported p values are two-sided, and statistical significance was defined as adjusted *p* < 0.05. Statistical significance was denoted as *p* < 0.05 (*), *p* < 0.01 (**), and *p* < 0.001 (***).

## Results

### Comprehensive single-cell atlas reveals liver microenvironment remodeling in cirrhosis

**Figure 4.**
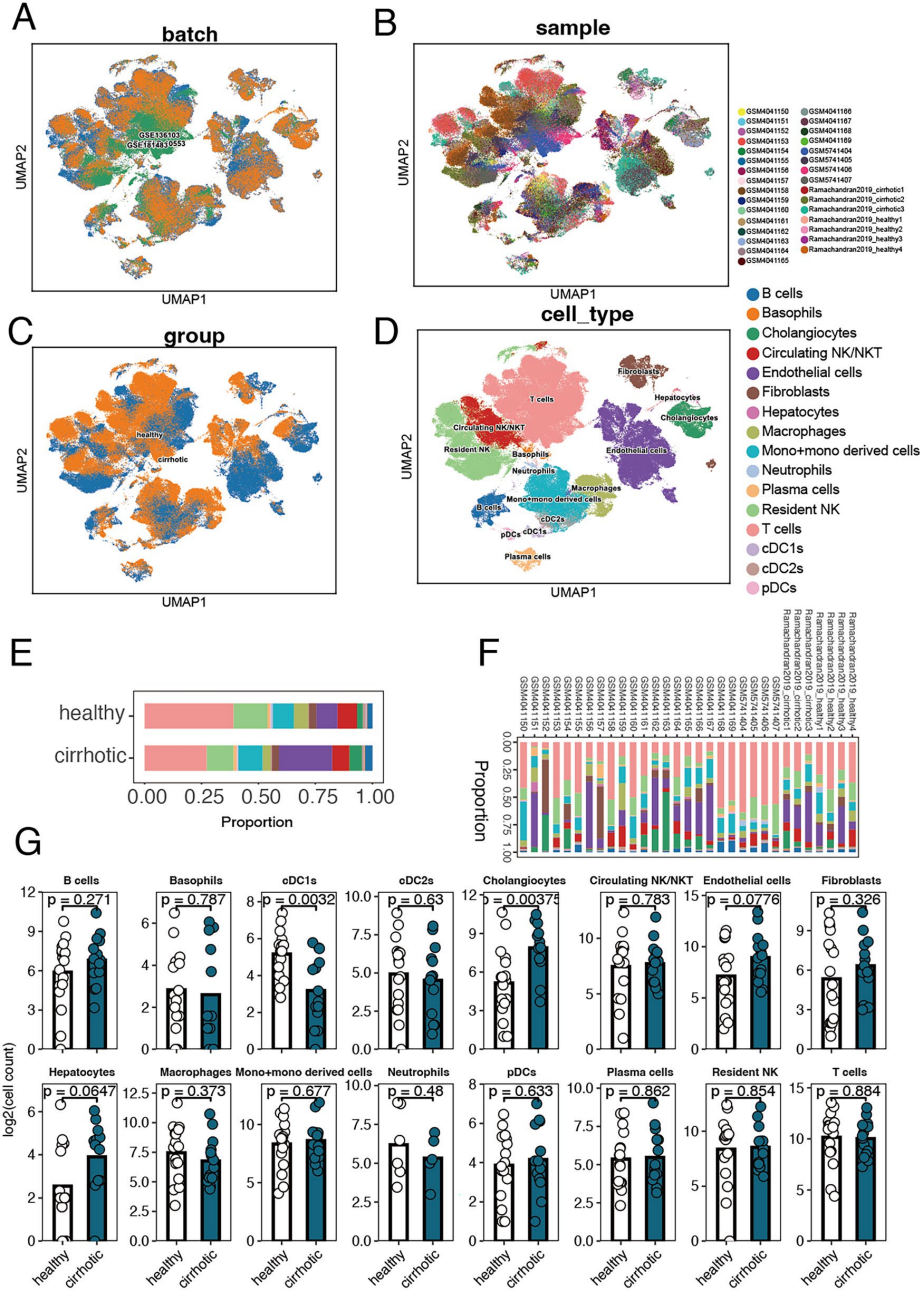
Single-cell atlas of 31 cirrhotic liver samples. (A) UMAP plot showing cell distributions across three batches (GSE136103, GSE181483, E-MTAB-10553); (B) UMAP plot showing cell distributions from 31 patient samples, including 17 healthy and 14 cirrhotic samples; (C). UMAP plot showing the distribution of cells between healthy and cirrhotic individuals; (D) Integrated UMAP plot annotated with 16 cell types, each colored by cluster; €Proportions of different cell types in healthy and cirrhotic samples; (F) Cell type proportions across all 31 samples; (G) Distribution of the 16 cell types in healthy vs. cirrhotic samples, with p-values indicated in the figure.

To construct a comprehensive atlas of the liver microenvironment in cirrhosis, we integrated single-cell RNA-sequencing data from 31 human liver samples, including 17 healthy controls and 14 patients with cirrhosis, across three publicly available datasets (GSE136103, GSE181483, and E-MTAB-10553). After quality control filtering, 224,529 cells were retained, ensuring a robust foundation for subsequent cell-type annotation and comparative analyses. Following batch correction with scVI, we obtained a unified UMAP embedding in which cells were well mixed across donors and datasets, indicating the successful removal of batch effects (Figure 4A,B). No obvious clustering by sample origin was observed, and cells from both healthy and cirrhotic samples broadly overlapped in reduced-dimensional space (Figure 4C).

Cell-type annotation using CellTypist identified 16 major populations, including immune subsets (T cells, B cells, macrophages, monocytes, NK/NKT cells, dendritic cells, plasma cells, and neutrophils) and structural cells (hepatocytes, cholangiocytes, fibroblasts, and endothelial cells) (Figure 4D). All the clusters contained cells from multiple individuals, confirming robust and consistent clustering across the dataset.

Next, we examined disease-associated changes in cellular composition. Compared to healthy livers, cirrhotic samples exhibited increased proportions of endothelial cells (23.49% vs. 9.25%) and cholangiocytes (5.58% vs. 2.57%), whereas immune cells such as macrophages (1.67% vs. 5.89%) and T cells (27.25% vs. 38.99%) were relatively reduced (Figure 4E). Similar patterns were observed at the per-sample level, further supporting diseaserelated remodeling of cell-type abundance (Figure 4F). Statistical testing confirmed that changes in specific populations, including endothelial cells and cholangiocytes, were significant (Figure 4G), reflecting an altered immune and stromal landscape in cirrhotic livers. Although not statistically significant, macrophages and T cells exhibited a decreasing trend in cirrhotic samples, suggesting potential immune shifts that merit further investigation.

### Suppressed programmed cell death pathways in macrophages from cirrhotic livers

**Figure 5.**
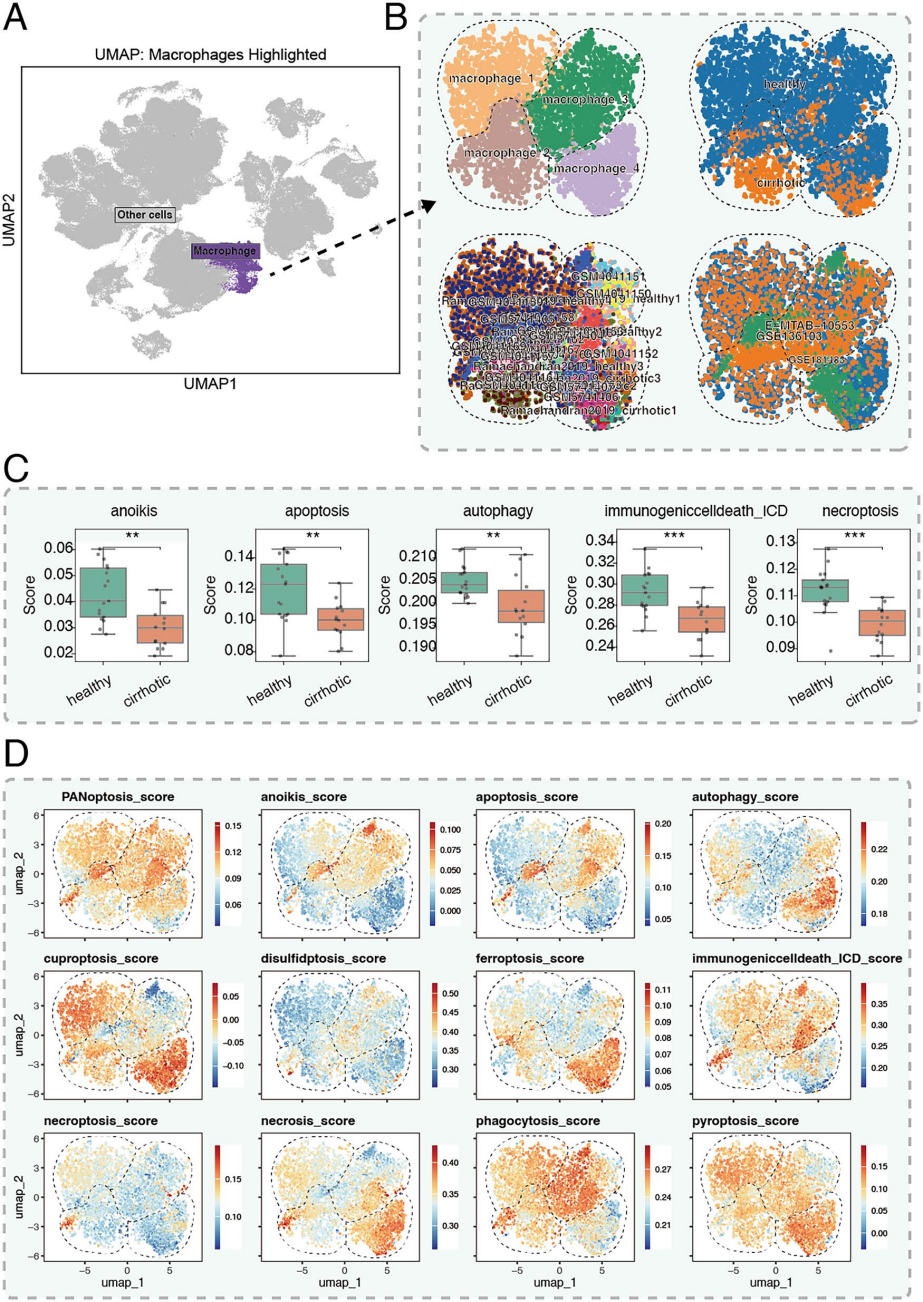
Basic characterization of macrophages and cell death scores across 31 samples. (A) UMAP plot showing integrated cells, with macrophages in purple and other cell types in grey; (B) UMAP plots showing four macrophage subclusters (top left), their distribution in healthy vs. cirrhotic tissues (top right), across 31 patients (bottom left), and across three batches (bottom right); (C) ssGSEA scores of five types of cell death in macrophages from healthy and cirrhotic samples (*p < 0.05, **p < 0.01, ***p < 0.001); (D) UMAP plot showing 12 types of cell death scores in macrophages; red indicates high scores, blue indicates low scores.

Macrophages showed a comparatively notable shift in proportion among immune cell populations, prompting further investigation of their role in cirrhosis. Based on the CellTypist annotation and cluster identity, cluster 9 was annotated as macrophages, comprising 4.0% of the total cells (Figure 5A). These cells formed a distinct immune subset within the UMAP space.

Unsupervised re-clustering revealed four transcriptionally distinct macrophage subsets (Figure 5B). Among them, Macrophage-1 and Macrophage-3 were predominantly healthy, while Macrophage-2 and Macrophage-4 included more cirrhotic-associated cells. The subset distribution was consistent across samples and batches, suggesting biological rather than technical variations.

To assess functional dysregulation in cirrhosis, we quantified the enrichment of the five cell death pathways using sample-level ssGSEA. Macrophages in cirrhotic livers showed significantly reduced enrichment for anoikis (*p* < 0.01), apoptosis (*p* < 0.01), autophagy (*p* < 0.01), immunogenic cell death (*p* < 0.001), and necroptosis (*p* < 0.001) (Figure 5C and Supplementary Figure S1). This consistent downregulation of multiple death pathways suggests global suppression of regulated cell death in cirrhotic macrophages.

To spatially contextualize these findings, we applied AUCell scoring of the 16 cell death pathways at single-cell resolution. Distinct regional enrichment patterns emerged: ferroptosis, necrosis, and autophagy were most active in Macrophage-4, while anoikis and apoptosis were predominant in Macrophage-3 (Figure 5D). In multiple pathways, including autophagy, necroptosis, and immunogenic cell death, cells from cirrhotic livers exhibited lower AUCell scores, mirroring the ssGSEA results and suggesting a cell state-specific suppression of death programs.

### Macrophage subsets show differential cell death suppression in cirrhosis

To further dissect functional heterogeneity among liver macrophages, we evaluated autophagy and necroptosis activation at single-cell resolution using AUCell scoring. While both pathways exhibited moderate variability across macrophage subpopulations, healthy-derived cells consistently displayed higher activation scores than those from cirrhotic livers (Figure 1A).

Applying global AUC thresholds (0.07 for autophagy, 0.05 for necroptosis) based on the Global_k1 strategy, we classified 22% and 20% of macrophages as pathway-activated for autophagy and necroptosis, respectively (Figure 1B). Visualization of the activation status revealed that active cells were predominantly located in healthy-enriched macrophage regions, with minimal representation in cirrhotic-derived areas (Figure 1C).

Assessment of macrophage subtype composition revealed a disease-associated redistribution: Macrophage-1 and Macrophage-2 were primarily found in healthy livers, while Macrophage-3 and Macrophage-4 were enriched in cirrhotic samples (Figure 1D). This pattern was consistently observed across individual patients (Figure 1D), suggesting that the macrophage subset composition was reshaped during disease progression.

Pathway activity varied significantly across the subtypes. Autophagy was highest in Macrophage-1 and lowest in Macrophage-4 (*p* < 0.001), whereas necroptosis peaked in Macrophage-3 and was lowest in Macrophage-4 (*p* < 0.001) (Figure 1E). These patterns matched the spatial activation observed in the AUCell plots (Figure 1A–C). Further stratification by disease group confirmed that autophagy scores were significantly reduced in cirrhosis (*p* = 0.0093), whereas necroptosis showed a non-significant downward trend (*p* = 0.14) (Figure 1F). Together, these findings point to the suppression of autophagy and necroptosis as early hallmarks of macrophage dysregulation in cirrhotic livers, particularly in disease-enriched subtypes.

To provide orthogonal tissue-level validation for the inferred macrophage death-state programs, we performed dual immunofluorescence staining on human liver sections. Consistent with the decreased autophagy activity suggested by AUCell/ssGSEA, LC3B signal within CD68-positive macrophages was significantly reduced in cirrhotic tissues, whereas p62/SQSTM1 showed an opposite increase, supporting impaired autophagic flux. In parallel, p-RIPK3 staining in CD68-positive macrophages was decreased in cirrhosis, corroborating suppression of necroptosis-related signaling. Representative images are shown in Figure 1G, with combined quantitative readouts summarized in Figure 1H.

### Macrophage differentiation trajectories reveal progressive loss of cell death activity

**Figure 6.**
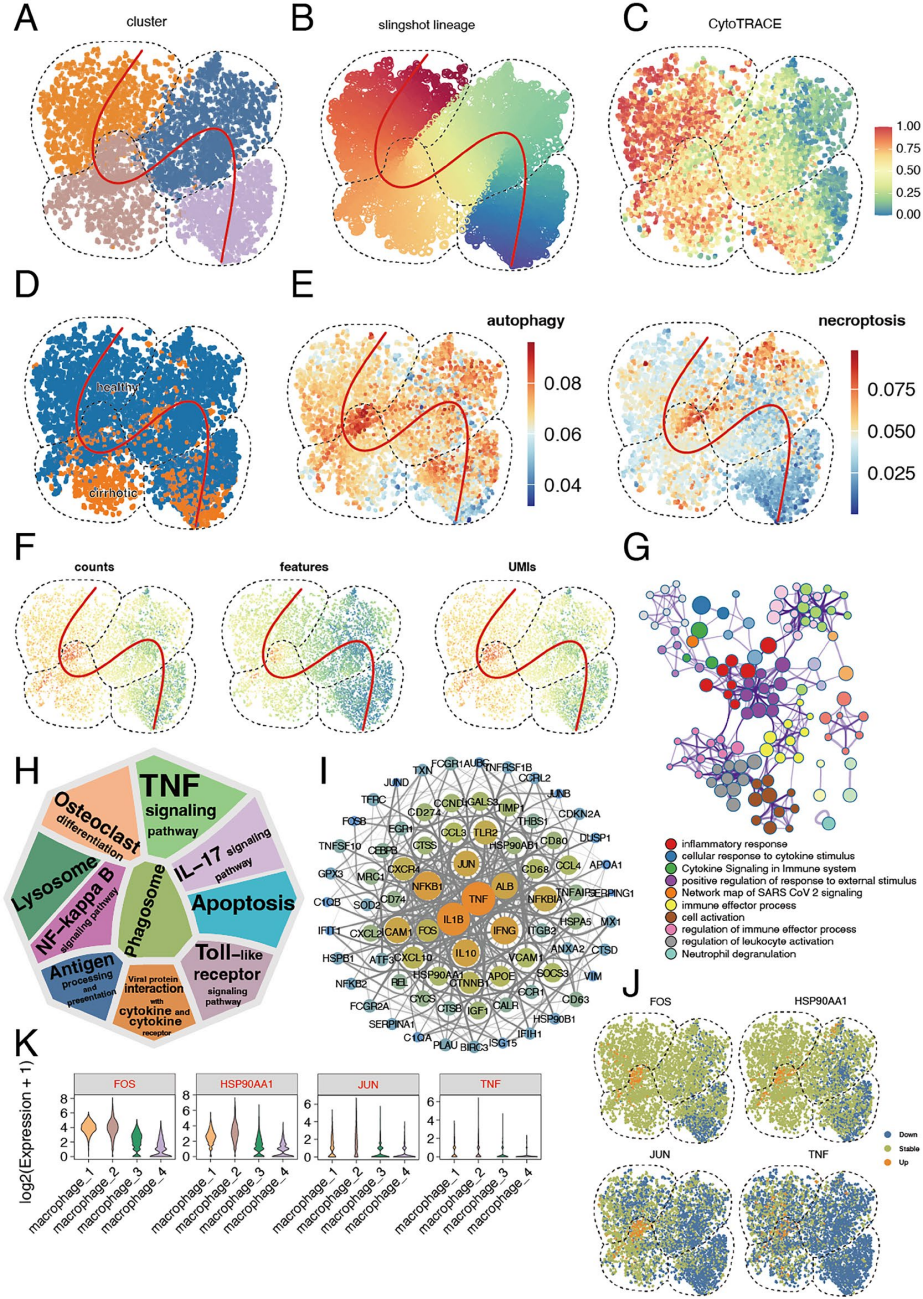
Macrophage trajectory and RNA velocity analysis, along with pseudotime-related gene identification. (A) UMAP plot showing macrophage pseudotime trajectories inferred by Slingshot; solid lines indicate lineages; (B) Pseudotime analysis with cells colored by their pseudotime value; darker blue indicates later stage; (C) UMAP plot colored by CytoTRACE scores showing macrophage differentiation potential; red indicates less differentiation, green indicates more mature cells; (D) Pseudotime analysis based on healthy vs. cirrhotic grouping; (E) AUCell scores of autophagy and necroptosis plotted along the pseudotime trajectory; scores decrease along the trajectory; (F) Expression level of total counts (left), number of expressed genes (middle), and number of marker genes (right) in each cell along pseudotime; red indicates higher values; (G) Generalized Additive Model (GAM) fitting using tradeSeq to identify genes significantly associated with pseudotime (FDR < E-16); GO enrichment of these genes performed using Metascape. Node size reflects gene number; color indicates functional cluster; (H) GO enrichment of dynamic genes; (I) Protein–protein interaction network of dynamic genes; (J) Expression levels of key pseudotime-related genes identified by CytoTRACE; red = high, blue = low, green = intermediate expression; (K) Expression dynamics of key pseudotime-related genes across the four macrophage clusters using CytoTRACE-based tradeSeq-GAM analysis.

To investigate whether the suppression of cell death pathways is linked to macrophage differentiation, we reconstructed pseudotime trajectories using Slingshot. Using Macrophage-1 as the root based on its CytoTRACE-derived position as the least differentiated macrophage population and its placement at the earliest stage of the inferred trajectory, a linear trajectory from Macrophage-1 to Macrophage-4 was inferred (Figure 6A). Pseudotime scores revealed a continuous transcriptional gradient from early to terminal states (Figure 6B), consistent with CytoTRACE analysis showing that Macrophage-1 harbored cells with high differentiation potential, while Macrophage-4 marked terminal states (Figure 6C).

When stratified by disease condition, healthy macrophages were predominantly localized at the early pseudotime, while cirrhotic cells were enriched in intermediate and terminal regions (Figure 6D). Notably, Macrophage-4 showed substantial cirrhotic enrichment, with an additional cirrhotic representation in Macrophage-2—suggesting either early exhaustion or redirection from canonical developmental paths.

Autophagy and necroptosis scores were elevated in early-stage macrophages (particularly Macrophage-2), and exhibited a progressive decline along the trajectory, reaching minimal levels in Macrophage-4 (Figure 6E). This pattern was mirrored by a steady decline in transcript complexity, including total RNA counts, gene numbers, and labeled markers (Figure 6F), indicating progressive loss of functional plasticity.

To identify genes associated with differentiation potential, we applied tradeSeq modeling using CytoTRACE scores as a continuous variable, with the number of knots set to 6 in the generalized additive model (GAM) framework. In total, 454 dynamic genes were identified (FDR < 1e–16). Both KEGG and Metascape-based GO enrichment analyses indicated that the majority of dynamic genes were associated with inflammatory-related processes, including leukocyte activation, cytokine signaling, and apoptosis (Figure 6G–H). These findings highlight the enrichment of immune-related pathways among genes associated with differentiation potential, suggesting that macrophage maturation involves coordinated transcriptional changes in immune functions.

Protein–protein interaction (PPI) network analysis identified central immune hub genes, including TNF, IL1B, NFKB1, IFNG, and IL10 (Figure 6I), which showed high connectivity within the interaction network and were also enriched in downstream immune-related modules.

In addition, several representative genes identified from the PPI network, including FOS, HSP90AA1, JUN, and TNF, exhibited gradual downregulation along pseudotime from high-potential to terminal macrophage states (Figure 6J,K), consistent with trajectory-based transcriptional attenuation. These genes showed a coherent decreasing expression pattern across the differentiation trajectory, with higher expression in early macrophage states and progressive reduction toward terminal states. Their expression dynamics are further shown in Supplementary Figure S2.

Collectively, these results suggest that immune regulatory programs gradually weaken during macrophage maturation in cirrhosis, accompanied by coordinated downregulation of genes associated with cellular stress and survival pathways, including autophagy- and necroptosis-related signaling components.

### Functional specialization of macrophage subtypes in cirrhosis

**Figure 7.**
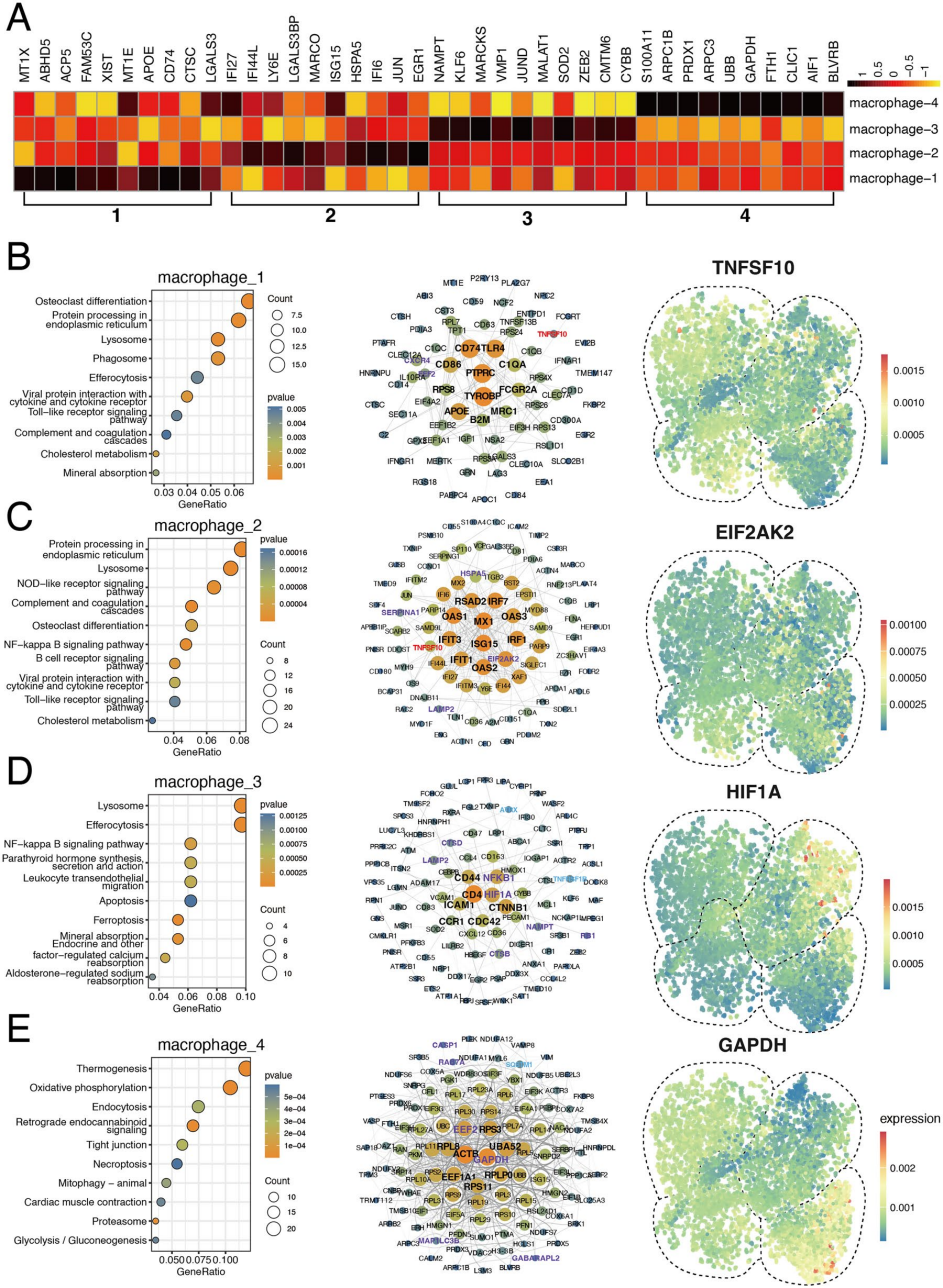
Functional analysis of four macrophage subclusters and identification of key marker genes. (A) Heatmap of marker gene expression across the four macrophage clusters; darker color indicates higher expression; (B) Macrophage-1: GO enrichment (left), interaction network (middle), and UMAP plot of marker gene expression (right). Autophagy-related genes in purple, necroptosis-related in blue, genes related to both in red; (C) Macrophage-2: Functional enrichment (left), interaction network (middle), and UMAP plot (right); (D) Macrophage-3: Functional enrichment (left), interaction network (middle), and UMAP plot (right); (E) Macrophage-4: Functional enrichment (left), interaction network (middle), and UMAP plot (right).

To further explore the functional landscape of macrophage heterogeneity, we performed cluster-specific analyses of gene expression, pathway enrichment, and interaction networks across four major macrophage subsets (Figure 7A–E, Figure S3–S6). The top marker genes for each cluster were identified using stringent criteria (min.pct > 0.25, |log2FC| > 0.25, FDR < 0.05) with no overlap among subsets, reflecting distinct transcriptional programs (Figure 7A).

Macrophage-1 showed strong innate immune and phagocytic signatures, including phagosome formation, TLR signaling, lysosomal activity, and efferocytosis (Figure 7B). Key hubs (TYROBP, FCGR2A, C1QA, TLR4) co-expressed autophagy-linked genes (CXCR4, EEF2). TNFSF10, involved in autophagy and necroptosis, was expressed in both Macrophage-1 and Macrophage-2.

Macrophage-2 was enriched for type I interferon and antiviral pathways (NOD, NF-κB, ER stress) (Figure 7C), with hub genes including MX1, ISG15, RSAD2. Autophagyrelated genes (EIF2AK2, HSPA5, LAMP2) suggested an IFN-driven stress-autophagy program.

Macrophage-3 showed enrichment of stress, migration, and cell death pathways (leukocyte migration, ferroptosis, apoptosis) (Figure 7D). Regulators included HIF1A, NFKB1, ICAM1, as well as autophagy and necroptosis genes (NAMPT, LAMP2, CTSB, TNFRSF1B, ATRX). HIF1A was most highly expressed here. The reduction of HIF1A may contribute to impaired mitophagy in cirrhotic macrophages, as HIF1A transcriptionally regulates key mitophagy mediators including BNIP3 and BNIP3L under hypoxic conditions [41–43].

Macrophage-4 displayed signatures of oxidative phosphorylation, mitophagy, glycolysis, and proteasome activity (Figure 7E). Hubs such as GAPDH, EEF2, ACTB, and autophagy genes (MAP1LC3B, GABARAPL2, RAB7A) defined a metabolically active, low-plasticity state. SQSTM1, regulating autophagy and necroptosis, was selectively expressed in this terminal subset.

Together, these findings reveal that macrophage subsets in cirrhosis represent a functional continuum from immune-responsive, autophagy-active populations to metabolically rewired, death-inactive states. The spatial validation of marker gene expression across the UMAP space is shown in Supplementary Figures S3–S6.

### Disease-specific remodeling of macrophage function and cell death programs

To identify cirrhosis-related transcriptional changes, we compared macrophages from healthy and cirrhotic livers (Figure 2A, Figure S7). Healthy macrophages expressed immune and redox genes (CXCL12, DUSP1, ZFP36, MT2A, MCL1), while cirrhotic macrophages upregulated interferon-stimulated genes (ISG15, IFI6, IFITM2) and lipid metabolism genes (PLTP, CETP) and downregulated key regulators (HIF1A, NAMPT, MT2A), consistent with pseudotime depletion.

Pathway enrichment of healthy markers revealed active immune signaling (NF-κB, TLR, apoptosis, phagosome maturation), aligning with early/intermediate subsets (Macrophage-1/2) (Figure 2B). To identify core regulators, we analyzed their PPI network, which highlighted immune and stress-related hubs (IL1B, TLR4, CXCR4, HIF1A, FOS), including autophagy genes (SQSTM1, HSP90AA1) (Figure 2C).

In contrast, cirrhotic macrophages showed enrichment in metabolic pathways (OXPHOS, cholesterol metabolism, vesicle trafficking) and loss of immune and death-related signatures (Figure 2D). Their PPI network was dominated by metabolic (GAPDH, ALB, APOE) and interferon genes (ISG15, MX1) with minimal autophagy nodes (Figure 2E). We next examined whether key components of the disease-associated macrophage marker program could be detected at the protein level *in situ*. Dual immunofluorescence staining showed increased CXCR4 and S100A10 signals within CD68-positive macrophages in cirrhotic livers compared with healthy controls (Figure 2F). The CD68-normalized quantification further confirmed the elevation of CXCR4/CD68 and S100A10/CD68 in cirrhosis (Figure 2G), supporting the translational relevance of the single-cell-derived markers.

Finally, Figures S8–S9 confirmed broad downregulation of autophagy and necroptosis genes (GABARAPL1, DAPK1, SQSTM1, MAP3K7) in cirrhosis, reinforcing the trajectory-based suppression of cell death (Figure 6) and the emergence of terminal, metabolically rewired macrophages.

### Cluster 3 of terminal macrophages exhibits disease-associated differentiation and diagnostic potential

To further dissect the heterogeneity within the terminal macrophage compartment, we reclustered Macrophage-4 into five subclusters using Seurat, resulting in well-balanced populations with distinct transcriptional signatures (Figure 3A). Slingshot trajectory inference identified two major lineages originating from cluster 4, which itself exhibited the highest CytoTRACE scores and were designated as the root state (Figure 3B). Among the inferred trajectories, lineage 2 closely mirrored the CytoTRACE-defined gradient and likely represents canonical terminal differentiation (Figure 3C). In contrast, lineage 1 traced a distinct path linking healthy to cirrhotic-enriched clusters, with cluster 3 selectively aligning along this disease-associated trajectory (Figure 3D).

CytoTRACE scores supported this directional model, indicating that cluster 4 was the least differentiated, while clusters 0 and 2 were the most mature (Figure 3C). Cluster 3 exhibited intermediate plasticity and a unique gene expression program that distinguished it from the other subclusters. Importantly, Cluster 3 was positioned within a pre-terminal ‘reprogramming window’ along the lineage, representing a transitional state prior to terminal fixation, where macrophages retain partial transcriptional plasticity and lineage adaptability.

Marker gene analysis revealed distinct transcriptional programs across the five sub-clusters (Figure 3E). Cluster 4 expressed TNFSF10, a dual regulator of autophagy and necroptosis, which is consistent with its pre-activation identity. Cluster 0 expressed metabolic and stress-response genes such as NAMPT and SOD2, consistent with a ter-minally reprogrammed state. Cluster 1 retained the autophagy marker (PPP1R15A), indicating intermediate activation. Importantly, cluster 3 was enriched for genes associated with ribosomal function, phagocytic activity, and immune modulation (e.g. LYZ, RNASE1, CORO1A, and S100A10), implicating it as a functionally distinct and pathologically relevant macrophage subset.

Functional enrichment of cluster 3 markers revealed a hybrid transcriptional pro-gram encompassing metabolic and immune features including oxidative phosphorylation, mitophagy, phagosome maturation, and ER protein processing (Figure 3F). The expression levels of key marker genes such as FXYD5, HCST, RGS10, and S100A6 were significantly elevated in cluster 3 and aligned with lineage 1 pseudotime dynamics, reinforcing its identity as a disease-altered transitional macrophage state. Given the potential link between CXCR4 and fibrosis-associated niche positioning, we further investigated the spatial relationship among CXCR4-positive cells, macrophages, and fibrotic septa in cirrhotic tissues. Using COL1A1 staining to delineate fibrotic septa, immunofluorescence revealed enrichment of CXCR4 signal in septa-adjacent regions as well as accumulation of CD68-positive macrophages along COL1A1-positive fibrotic tracts (Figure 3H). Quantitative spatial analysis demonstrated a decreased relative distance of CXCR4-positive cells to fibrotic septa together with an increased density of CD68-positive macrophages in septa-proximal areas in cirrhosis (Figure 3I). Collectively, these results connect the CXCR4-associated macrophage program to a fibrosis-linked microanatomical niche.

**Figure 8.**
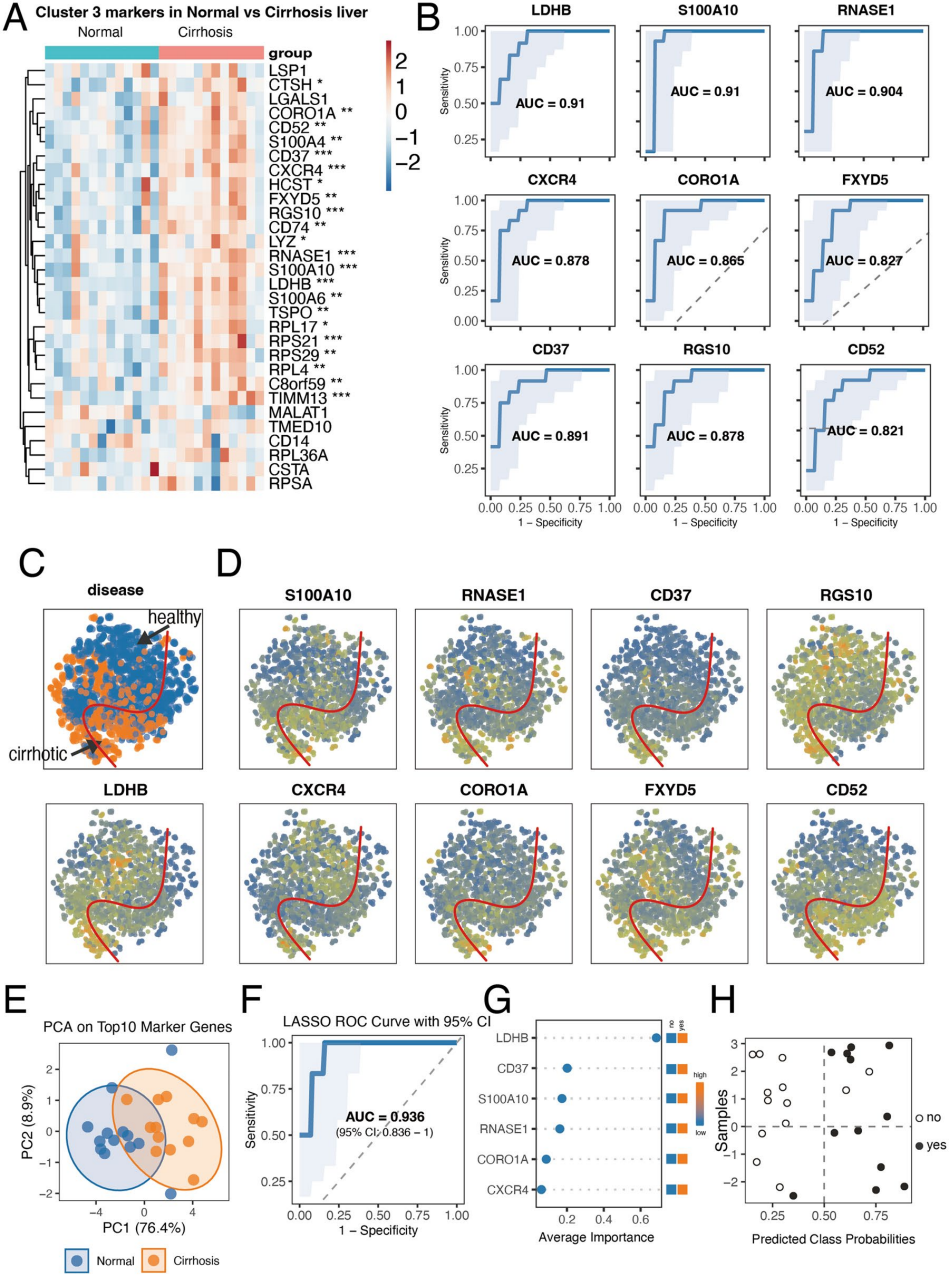
Validation of key marker genes in bulk RNA-seq dataset (GSE89377). (A) Validation of Macrophage-4 cluster 3 marker genes in GSE89377; red = upregulated, blue = downregulated (healthy vs. cirrhotic); (B) ROC validation of cluster 3 marker genes; (C) UMAP plot showing Slingshot differentiation trajectories and gene expression patterns between healthy and cirrhotic samples; solid line = lineage 1; (D) UMAP plots showing marker gene expression and Slingshot trajectories; blue = low, yellow = medium, red = high expression; (E) Diagnostic efficiency for cirrhosis vs. non-cirrhosis using the top 10 significant genes; (F) ROC curve of 6-factor predictive model including key metabolites; (G) Genes included in the 6-factor prediction model; (H) Confusion matrix showing the prediction performance of the 6-factor model for cirrhosis and healthy classification.

To assess the translational relevance of cluster 3 markers, we evaluated their expression in an independent bulk liver transcriptome dataset (GSE89377; 13 healthy and 12 cirrhotic samples). Most of the top 25 marker genes from cluster 3 showed significantly higher expression in cirrhotic samples (Figure 8A). Several genes—such as LDHB, RNASE1, S100A10, and CD37—demonstrated strong discriminative power with AUC values ranging from 0.82 to 0.91 in ROC analyses (Figure 8B).

When mapped onto single-cell Slingshot lineage 1, these marker genes exhibited increased expression along the healthy-to-cirrhotic trajectory, further supporting their association with pathologic macrophage differentiation (Figure 8C–D). A subset of the top-ranked marker genes was used to construct a diagnostic model *via* LASSO regression. The resulting 6-gene signature, LDHB, CD37, S100A10, RNASE1, CXCR4, and CORO1A, achieved high predictive performance (AUC = 0.936; 95% CI: 0.836–1.000), with near-perfect classification accuracy between healthy and cirrhotic samples (Figure 8E–H). Notably, CXCR4 has been reported as a regulator of autophagy, further linking this disease-associated signature to the suppression of death-related pathways.

Together, these analyses uncovered a coordinated suppression of autophagy and necroptosis in liver macrophages during cirrhosis. Among these, Macrophage-4 cluster 3 emerges as a transcriptionally distinct, disease-enriched subpopulation that embodies the key molecular hallmarks of cirrhotic remodeling, namely metabolic adaptation, immune attenuation, and loss of death-related plasticity. Importantly, the expression of cluster 3 marker genes was robustly validated using bulk transcriptomic data and demonstrated potential diagnostic value.

These findings suggest that macrophage-driven modulation of programmed cell death pathways, especially autophagy and necroptosis, may play dual roles in cirrhosis progression, which may enable terminal macrophage persistence in fibrotic tissue while compromising immune clearance and repair. Further investigation of these pathways may reveal therapeutic avenues to either reverse maladaptive macrophage programming or reengage beneficial death mechanisms in chronic liver disease.

## Discussion

Chronic liver disease is increasingly recognized as an immunometabolic disorder characterized by profound immune dysregulation and metabolic remodeling, which drive progressive tissue remodeling. Here, we present a high-resolution single-cell atlas of the human liver immune microenvironment under healthy and cirrhotic conditions, integrating over 200,000 cells from 31 samples. Among 16 identified cell types, macrophages emerged as a transcriptionally pivotal population reshaped in cirrhosis.

A key discovery is the coordinated suppression of autophagy, necroptosis, apoptosis, and immunogenic cell death in cirrhotic macrophages. This death-resistant phenotype aligns with a structured differentiation trajectory, where early-stage, immune-active macrophages shift toward metabolically rewired, immune-inert terminal states. Pseudotime and CytoTRACE analyses revealed declining transcriptomic complexity, differentiation potential, and downregulation of immune regulators such as TNF, IL1B, and HIF1A, which are closely linked to autophagy and cell death regulation [44].

Importantly, we found that autophagy and necroptosis scores dropped sharply along the macrophage trajectory, with Macrophage-4 showing minimal activation. This supports a model where death suppression represents an active, disease-adaptive state. Similar resistance phenotypes have been reported in fibrotic and tumor-associated macrophages [45]. The fact that autophagy suppression tracks both subtype identity and disease stage underscores its potential role as an early and sustained hallmark of pathological macrophage remodeling. Importantly, our immunofluorescence validation provides protein-level support for these computational inferences. The decrease in LC3B and p-RIPK3 signals together with the accumulation of p62/SQSTM1 within CD68-positive macrophages in cirrhotic tissues mirrors the directionality of the single-cell pathway scores (Figure 1G,H). This orthogonal evidence strengthens the conclusion that terminal macrophage states in cirrhosis are characterized by coordinated attenuation of autophagy and necroptosis programs.

At the molecular level, this reprogramming is characterized by the downregulation of canonical autophagy mediators (e.g. MAP1LC3B, SQSTM1), necroptosis regulators (e.g. DAPK1), and upstream stress sensors (e.g. HIF1A, NAMPT), many of which are embedded in larger immune and metabolic networks. These alterations suggest that macrophage survival in the cirrhotic niche is sustained by suppression of stress-adaptive death programs, thereby facilitating prolonged tissue residency, fibrotic persistence, and impaired immune clearance. Notably, recent work has shown that myeloid-specific deletion of autophagy genes, such as Atg5 or Atg7, in murine models results in exaggerated inflammation and fibrosis, further validating the immunomodulatory role of death pathways in hepatic macrophages [46].

In addition to intrinsic macrophage programs, the clearance of apoptotic bodies by neighboring non-parenchymal cells, including Kupffer cells and hepatic stellate cells, represents an important layer of regulation in the hepatic injury microenvironment. Previous work has shown that efficient engulfment of apoptotic hepatocytes is critical for the resolution of inflammation, whereas impaired efferocytosis can promote secondary necrosis and amplify profibrotic signaling in the liver [47,48]. In the context of cirrhosis, persistent inflammatory and metabolic stress may further compromise this clearance system, thereby reinforcing a profibrotic niche. Together, these observations indicate that impaired macrophage cell death and defective clearance of apoptotic cells may act in concert to sustain a pro-fibrotic hepatic microenvironment.

Trajectory inference confirmed a smooth, disease-skewed progression from Macrophage-1/2 to Macrophage-4, paralleling observations in chronically inflamed or tumor environments [49]. Downregulation of HSP90AA1, FOS, and JUN related transcriptional stress integrators across pseudotime further reflects the loss of immune plasticity. Interestingly, several of these genes also play central roles in cell death sensitization and immune checkpoint regulation [50]. The convergence of these programs suggests global attenuation of macrophage plasticity in favor of metabolic survival, consistent with the energy-constrained, hypoxic, and cytokine-rich milieu of fibrotic livers [51].

The heterogeneity of hepatic macrophages in cirrhosis extends beyond changes in the population proportion or pathway activity, reflecting profound transcriptional and functional rewiring along a continuum of differentiation. Together, these macrophage subsets form a continuum from homeostatic to terminally reprogrammed states. Subclustering and functional profiling revealed that each macrophage subset (Macrophage-1 to −4) was characterized by distinct pathway signatures and gene networks. Notably, Macrophage-1, enriched in healthy livers, retains potent innate immune functions, phagocytic capacity, and robust autophagy signaling, resembling restorative monocyte-derived macrophages in regeneration [52]. Macrophage-2 appears to represent an intermediate inflammatory-activated state, characterized by interferon-response signaling and stress adaptation, likely reflecting early immune reprogramming under chronic inflammatory pressure prior to terminal dysfunction.

In contrast, Macrophage‑4 exhibits metabolic specialization marked by increased oxidative phosphorylation and glycolytic flux, along with selective retention of mitophagy-related programs. Expression of MAP1LC3B and GABARAPL2 suggests mitochondrial autophagy supports bioenergetic adaptation, consistent with prior findings that OXPHOS-dependent macrophage polarization retains mitophagic capacity to preserve mitochondrial homeostasis [53].

Of particular interest is the identification of Macrophage-4 cluster 3 as a disease-associated subpopulation with unique transcriptional identity and diagnostic potential. This subset, which occupies an intermediate pseudotime position, represents a transitional macrophage state characterized by integrated immune modulation, metabolic adaptation, and altered tissue-interaction programs rather than a marker-driven entity. Several of these genes have been implicated in macrophage activation, phagocytosis, and tumor immune evasion, supporting their functional relevance in pathological remodeling [54]. The expression dynamics of cluster 3 markers along the cirrhosis-associated lineage trajectory reinforce its role as a transitional intermediate enriched during disease progression.

Importantly, these transcriptional markers retained high discriminatory power in bulk liver transcriptome data, with ROC AUCs exceeding 0.9 for genes such as LDHB, S100A10, and RNASE1. Furthermore, the six-gene LASSO diagnostic signature achieved an AUC of 0.936 (95% CI: 0.836–1.000), demonstrating strong discriminatory capacity between healthy and cirrhotic samples. This highlights the translational relevance of our single-cell atlas, which offers a molecular fingerprint that could inform diagnostic or stratification strategies in clinical settings. The inclusion of CXCR4, a dual autophagy and chemotaxis regulator, in the six-gene diagnostic model further emphasizes the coupling of immune positioning and regulation of the death program in cirrhotic macrophages [55]. These findings support the development of non-invasive biomarkers or targeted immunotherapies that harness disease-enriched macrophage states. In addition, the tissue-level elevation of CXCR4 and S100A10 within CD68-positive macrophages (Figure 2F–G), together with their fibrosis-linked spatial patterning relative to COL1A1-positive septa (Figure 3H–I), suggests that the identified marker program is not only transcriptionally enriched but also manifests as a niche-associated phenotype *in situ*. This supports a model in which disease-associated macrophages progressively acquire a septa-proximal, survival-reprogrammed state that may influence fibrotic remodeling through altered immune crosstalk and persistence. These findings further suggest that septa-associated macrophages may function as niche-localized drivers of fibrotic scarring during cirrhosis progression. If serial sections were used for part of the spatial analysis, future work using multiplexed staining or spatial transcriptomics will further refine cell-type-resolved co-localization and neighborhood relationships.

Together, our data propose a mechanistic framework whereby macrophage differentiation in cirrhosis is coupled with the progressive suppression of regulated cell death, diminished immune plasticity, and metabolic adaptation. These features may collectively promote macrophage persistence within fibrotic tissue, while potentially limiting immune surveillance, tissue remodeling, and pathogen clearance. This form of persistence resembles the macrophage programming observed in other chronic fibrotic and neoplastic contexts, including idiopathic pulmonary fibrosis and tumor-associated macrophage infiltration [56]. Taken together, these findings suggest that the death- suppression trajectory may represent a universal hallmark of chronic fibrotic diseases.

Our findings add a critical layer to the growing literature on hepatic immune reprogramming in chronic liver diseases. Previous single-cell studies have primarily focused on defining cell identities and mapping inflammatory trajectories in liver resident cells [57], but have paid less attention to how macrophage differentiation is coupled to regulated cell death programs and functional immune decline in human cirrhosis.

In this study, we integrate differentiation trajectory analysis with dynamic changes in autophagy and necroptosis, together with spatial validation and diagnostic modeling. This approach builds upon prior murine studies showing that impaired macrophage apoptosis and efferocytosis promote fibrosis progression, and further identifies a disease-associated transitional macrophage state with septa-proximal localization and clinical relevance, providing new insight into macrophage remodeling in cirrhosis [19].

A key translational insight from this study is the identification of disease-enriched macrophage intermediates, particularly cluster 3 of Macrophage-4, where suppression of death programs appears at the pre-terminal differentiation stages, suggesting a therapeutic window prior to terminal macrophage fixation. Supporting this, pharmacological restoration of autophagic or necroptotic competence has shown promise in preclinical models of liver fibrosis and hepatocellular carcinoma [58]. Furthermore, the discovery of a robust six-gene diagnostic signature, including CXCR4 and S100A10, has provided potential biomarkers for disease stratification. Although CXCR4 is well known for its role in hepatic immune cell trafficking [59], our data highlight its value as a surrogate marker for autophagy-linked macrophage states. This immune state-informed signature provides a foundation for more dynamic and functional cirrhosis diagnostics. From a systems perspective, our integrative analysis suggests that macrophage persistence in cirrhosis arises from coupled transcriptional and metabolic reprogramming, which prioritizes immune suppression and survival over clearance. This is consistent with the concept of trained tolerance in chronically stressed tissues, where sustained adaptation to injury becomes maladaptive, promoting fibrotic progression and immune evasion [60].

Although our findings provide a high-resolution map of macrophage reprogramming in cirrhosis and offer mechanistic insights into cell death suppression and metabolic adaptation, several limitations should be acknowledged. Among them is the reliance on publicly available single-cell RNA-sequencing datasets, which may insufficiently capture rare macrophage subsets or early-stage disease transitions. Moreover, the functional roles of the key transcriptional programs and diagnostic markers identified here remain to be validated *in vivo*. Future studies incorporating spatial transcriptomics, proteomic profiling, and longitudinal sampling will be essential to confirm and expand these observations.

## Conclusion

In this study, we constructed a high-resolution single-cell atlas of human liver immune cells in healthy and cirrhotic conditions and systematically characterized macrophage heterogeneity. Using pseudotime trajectory inference, we demonstrated that macrophages undergo a continuous differentiation process from early immune-active states to terminal metabolically rewired states in cirrhosis. Integrative analysis of pathway activity revealed a progressive and coordinated suppression of autophagy, necroptosis, apoptosis, and immunogenic cell death along this trajectory. Notably, key regulators of these processes, including RIPK3 and related cell death-associated molecules, were identified as central components of the observed transcriptional programs.

Furthermore, we identified a disease-associated transitional macrophage population characterized by septa-proximal localization and distinct transcriptional programs. Through integrated trajectory inference, histological spatial validation, and machine-learning–based modeling, we established a macrophage-derived diagnostic gene signature that robustly discriminates between cirrhotic and healthy samples.

Together, our findings define a previously unrecognized macrophage differentiation cell death axis in cirrhosis and provide mechanistic insight into macrophage reprogramming as well as potential translational biomarkers for chronic liver disease. Future studies incorporating spatial transcriptomics, functional validation of key macrophage subpopulations, and macrophage-targeted interventions or drug delivery strategies with potentially efficacious targets will be essential to further validate and translate these findings into clinical applications.

## Supplementary Material

Supplemental Material

## Data Availability

All the data analyzed in this study were obtained from publicly available repositories. Single-cell RNA-sequencing (scRNA-seq) data from 31 adult human liver samples (including 17 healthy individuals and 14 patients with liver cirrhosis) were retrieved from the Gene Expression Omnibus (GEO) and ArrayExpress databases under accession numbers GSE136103, GSE181483, and E-MTAB-10553.
