# Supplementary material for: Progressive suppression of regulated cell death defines terminal macrophage states in liver cirrhosis
Source: Ann Med. 2026 Jun 25;58(1):2690717. doi: 10.1080/07853890.2026.2690717 (PMC13307378; doi:10.1080/07853890.2026.2690717)
Supplement: Supplemental Material [file IANN_A_2690717_SM4886.zip › suppl_data/Suppl_fig, table caption.docx]

**Supplementary** **Figures**

Figure.S1. ssGSEA statistics of 16 cell types, cell death pathways, and inter-sample comparison.

Figure.S2. Expression dynamics of pseudotime-correlated genes in macrophages; lighter color = higher value.

Figure.S3–S6. Marker gene distributions in Macrophage-1 to -4; red = higher expression.

Figure.S7. Violin and UMAP plots of significantly downregulated genes in cirrhotic samples.

Figure.S8. Differential expression of autophagy-related genes between healthy and cirrhotic samples.

Figure.S9. Differential expression of necroptosis-related genes between healthy and cirrhotic samples.

**Supplementary Tables**

Table S1. Source publications for the three datasets.

Table S2. Background gene sets of the 12 cell death types.
