# Supplementary figures and images for: Progressive suppression of regulated cell death defines terminal macrophage states in liver cirrhosis
Source: Ann Med. 2026 Jun 25;58(1):2690717. doi: 10.1080/07853890.2026.2690717 (PMC13307378; doi:10.1080/07853890.2026.2690717)

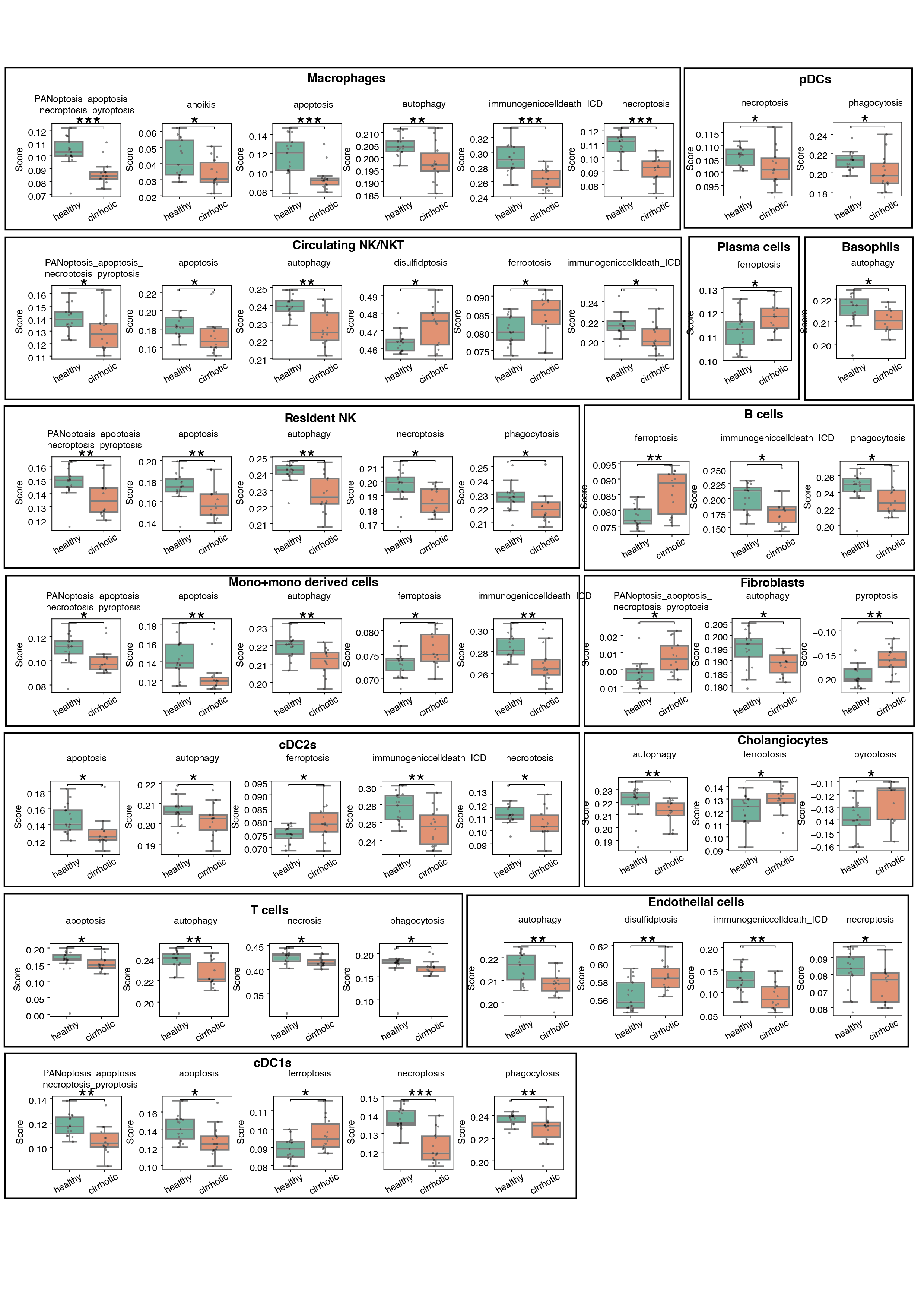

Supplement: Supplemental Material [file IANN_A_2690717_SM4886.zip › suppl_data/FigS1.png]

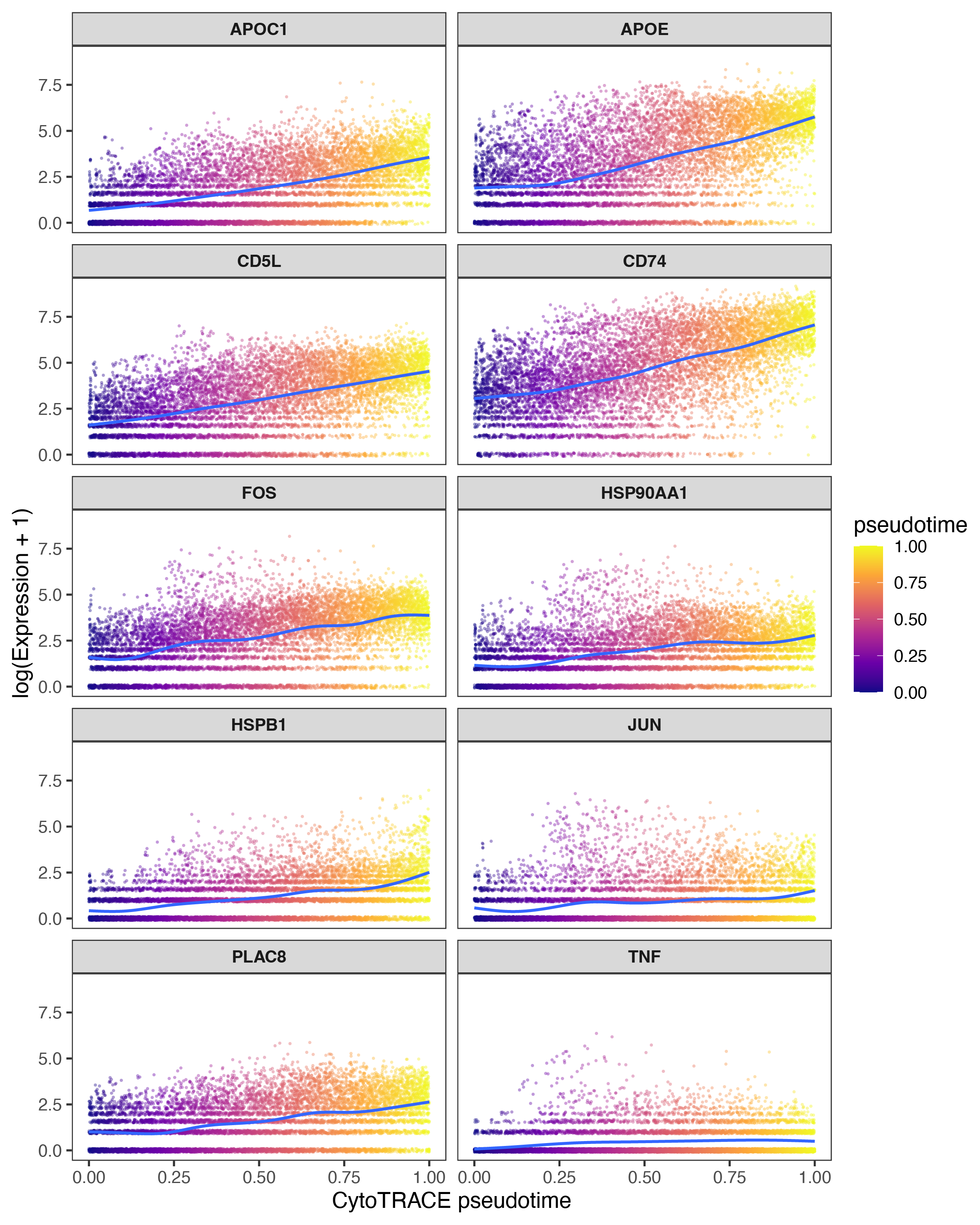

Supplement: Supplemental Material [file IANN_A_2690717_SM4886.zip › suppl_data/FigS2.png]

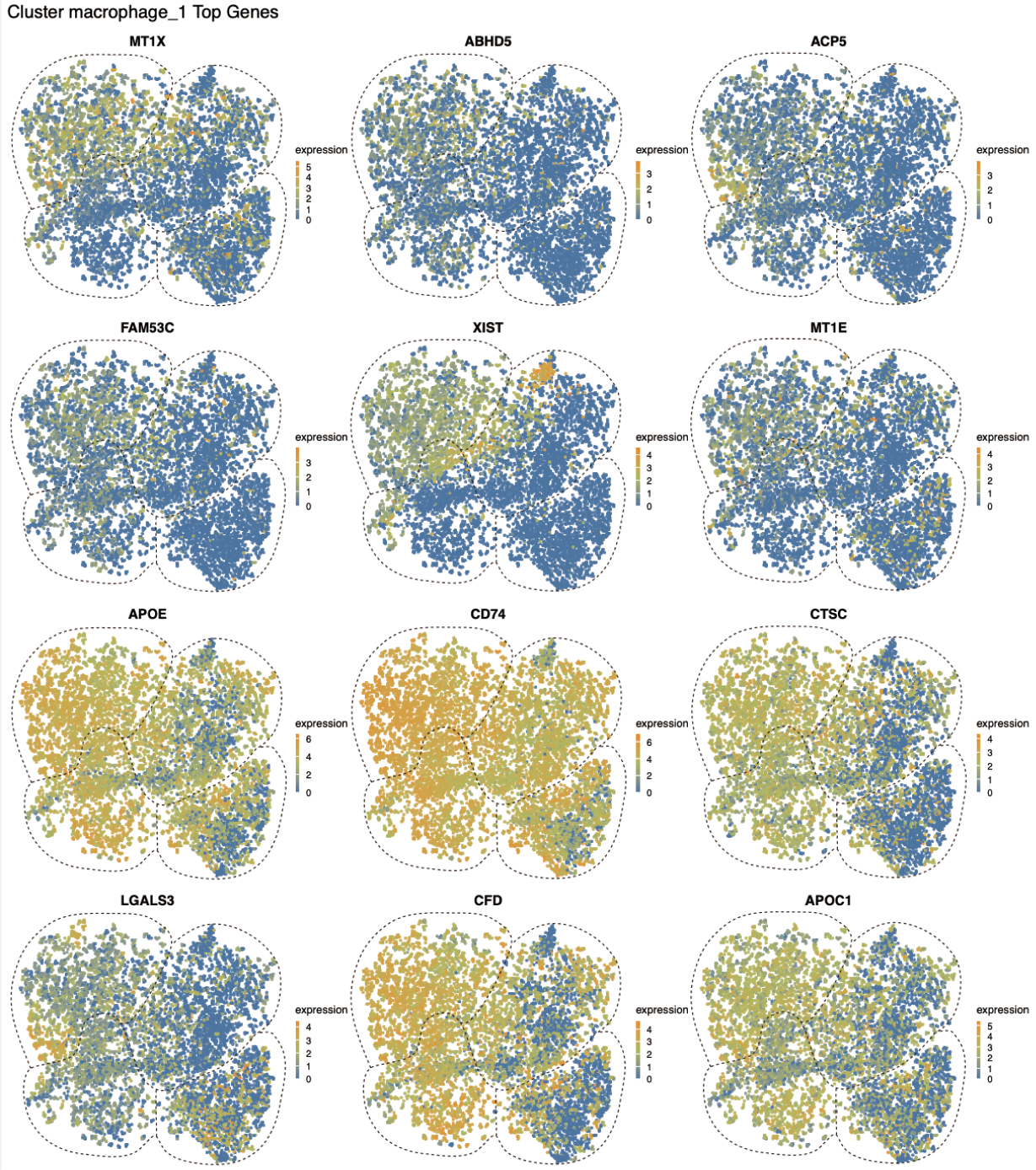

Supplement: Supplemental Material [file IANN_A_2690717_SM4886.zip › suppl_data/FigS3.png]

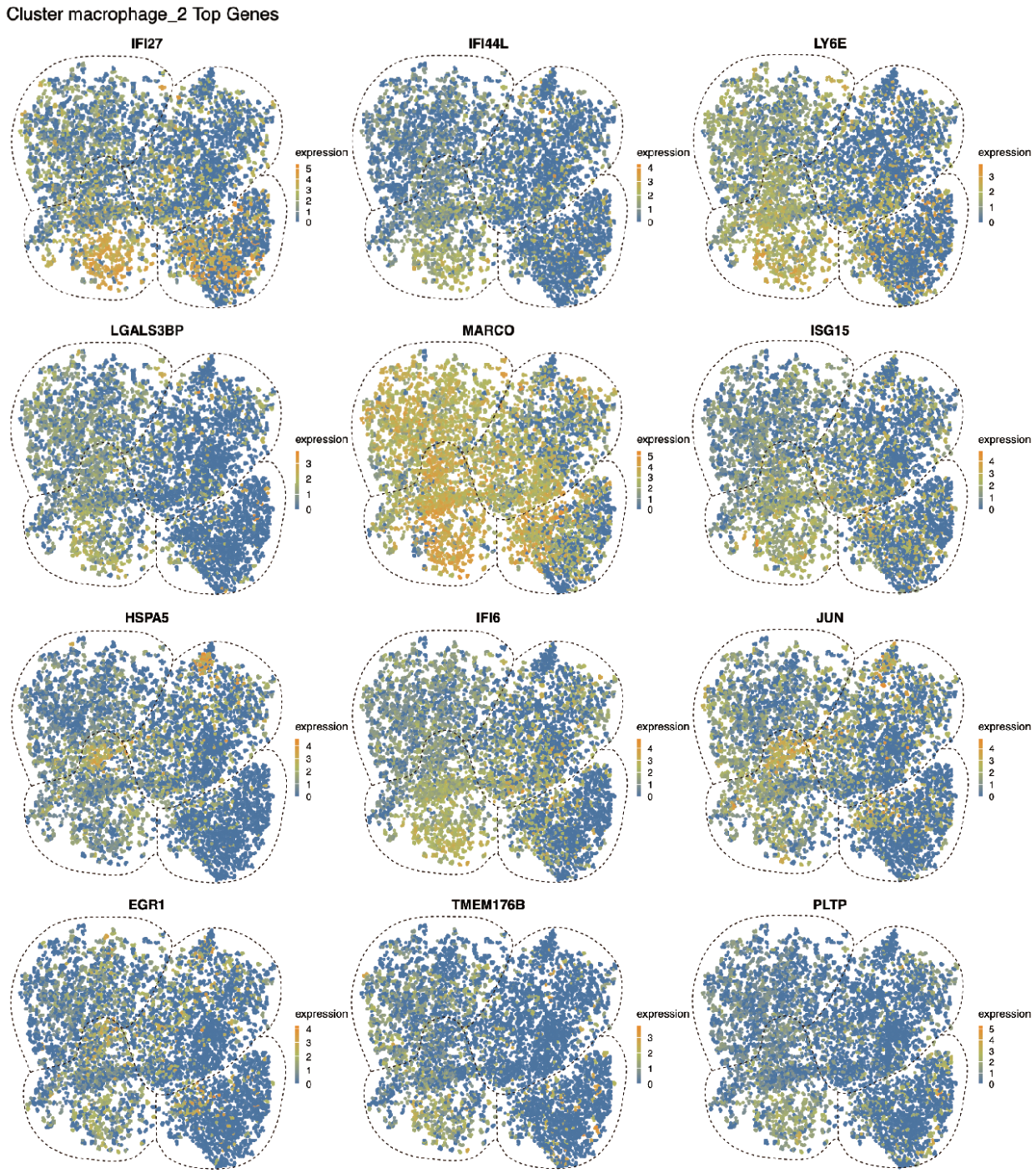

Supplement: Supplemental Material [file IANN_A_2690717_SM4886.zip › suppl_data/FigS4.png]

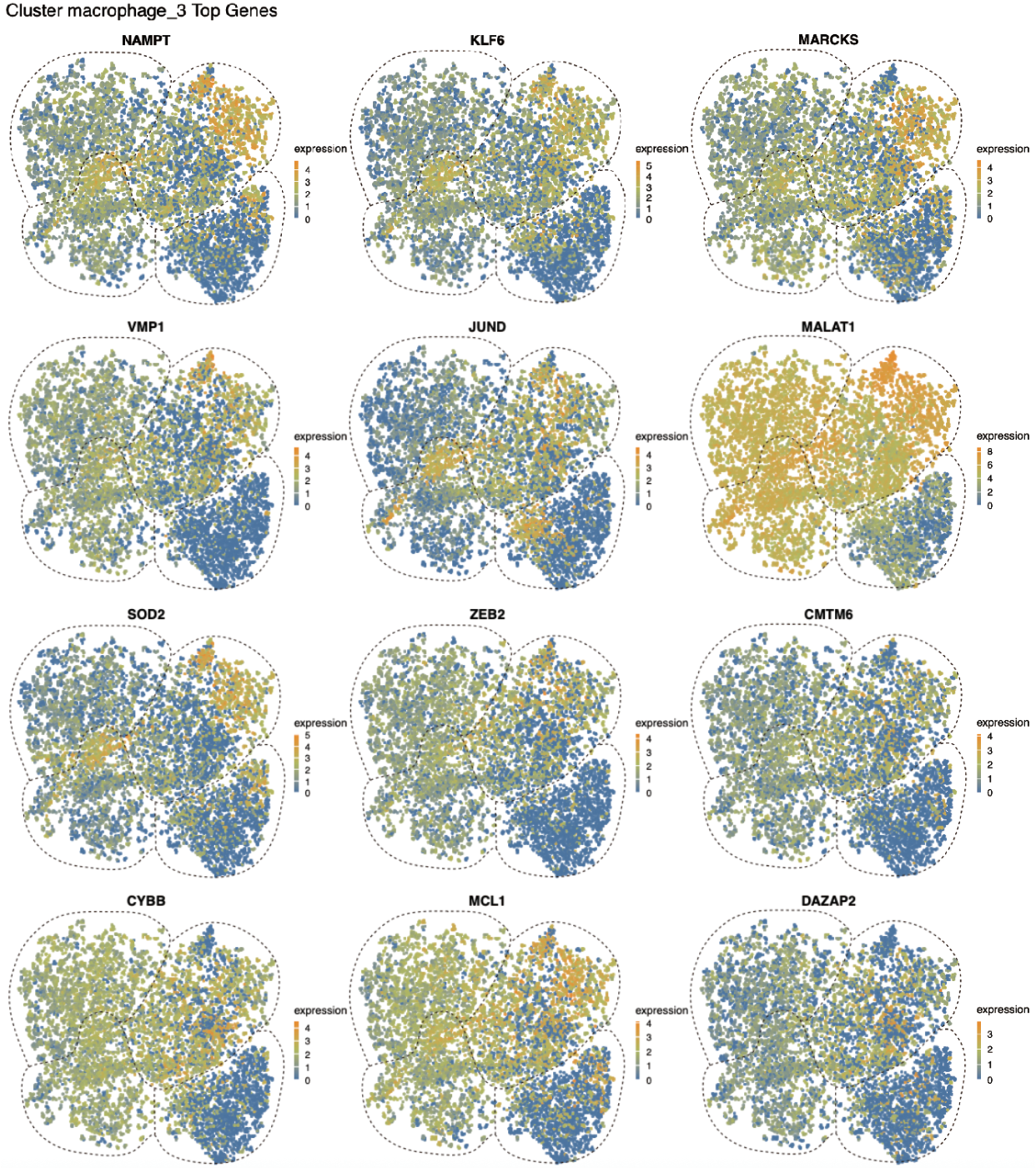

Supplement: Supplemental Material [file IANN_A_2690717_SM4886.zip › suppl_data/FigS5.png]

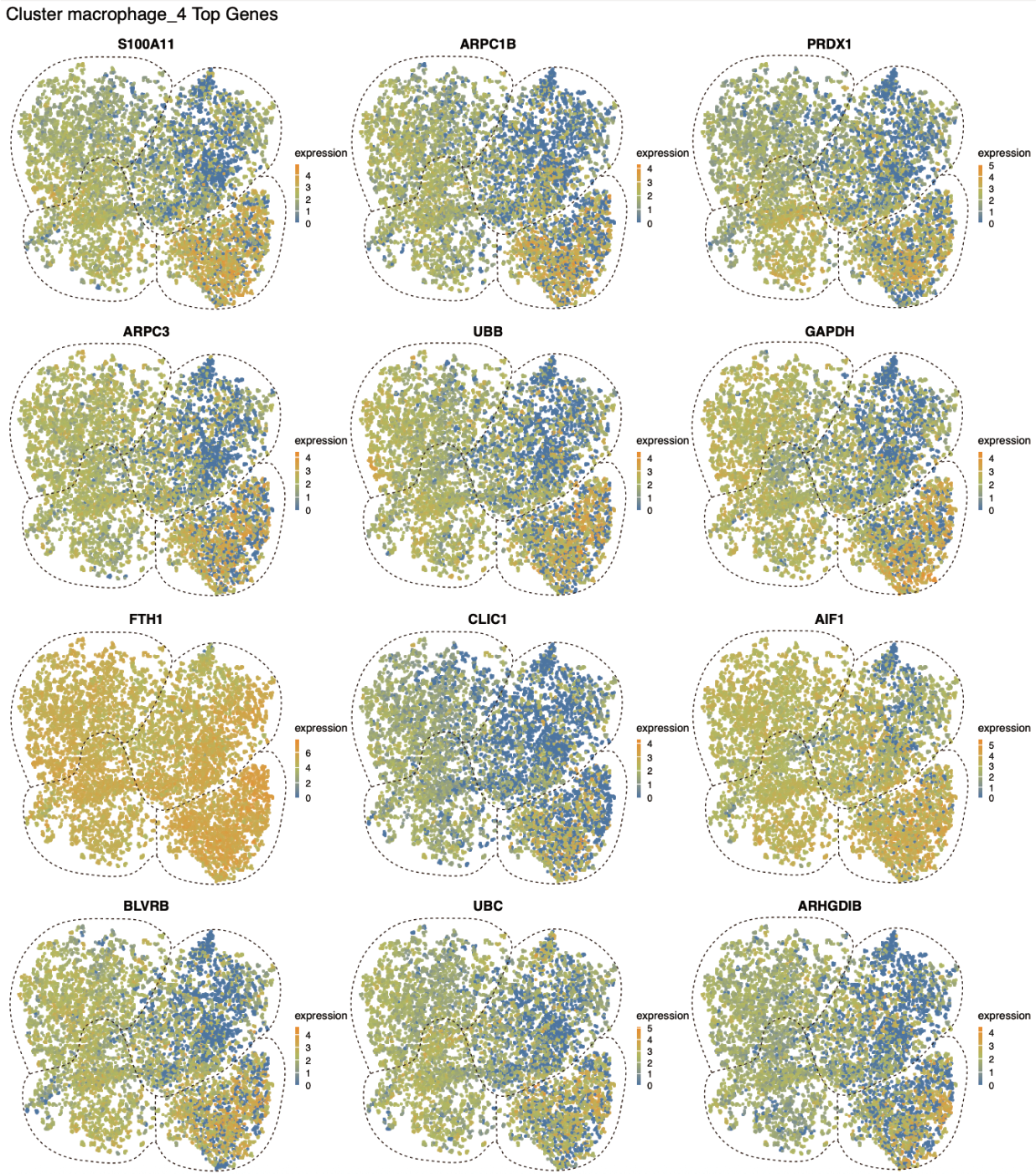

Supplement: Supplemental Material [file IANN_A_2690717_SM4886.zip › suppl_data/FigS6.png]

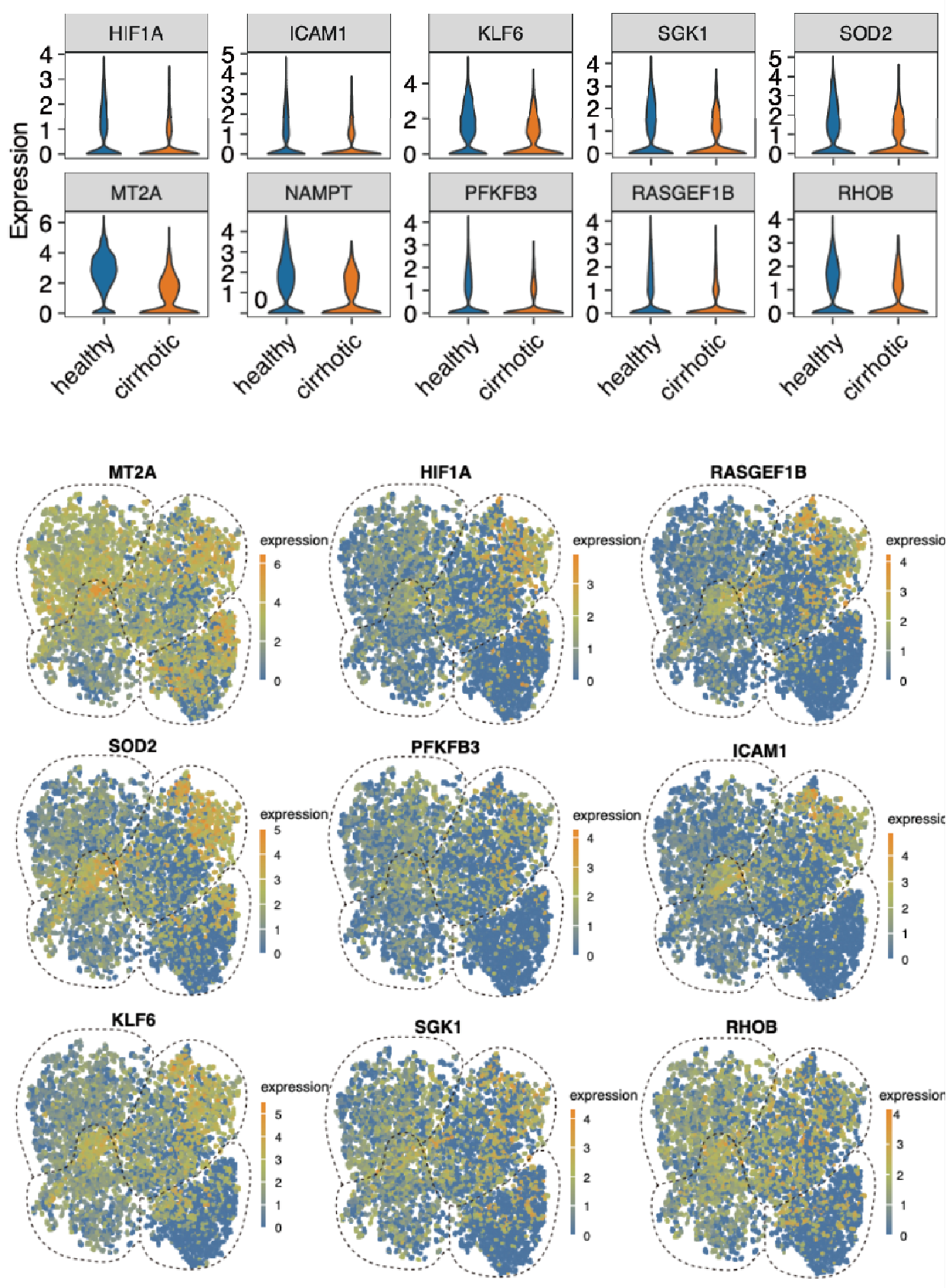

Supplement: Supplemental Material [file IANN_A_2690717_SM4886.zip › suppl_data/FigS7.png]

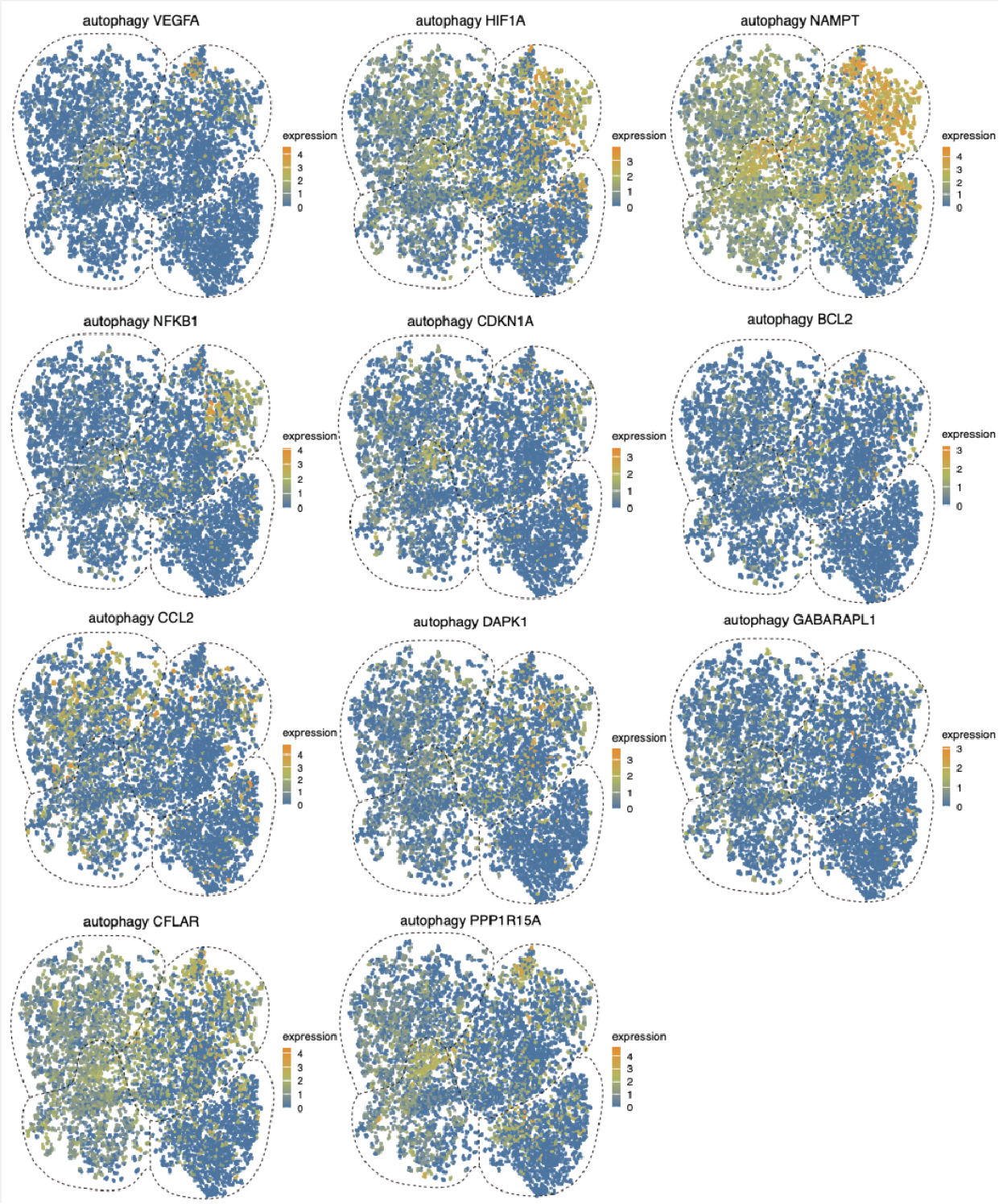

Supplement: Supplemental Material [file IANN_A_2690717_SM4886.zip › suppl_data/FigS8.png]

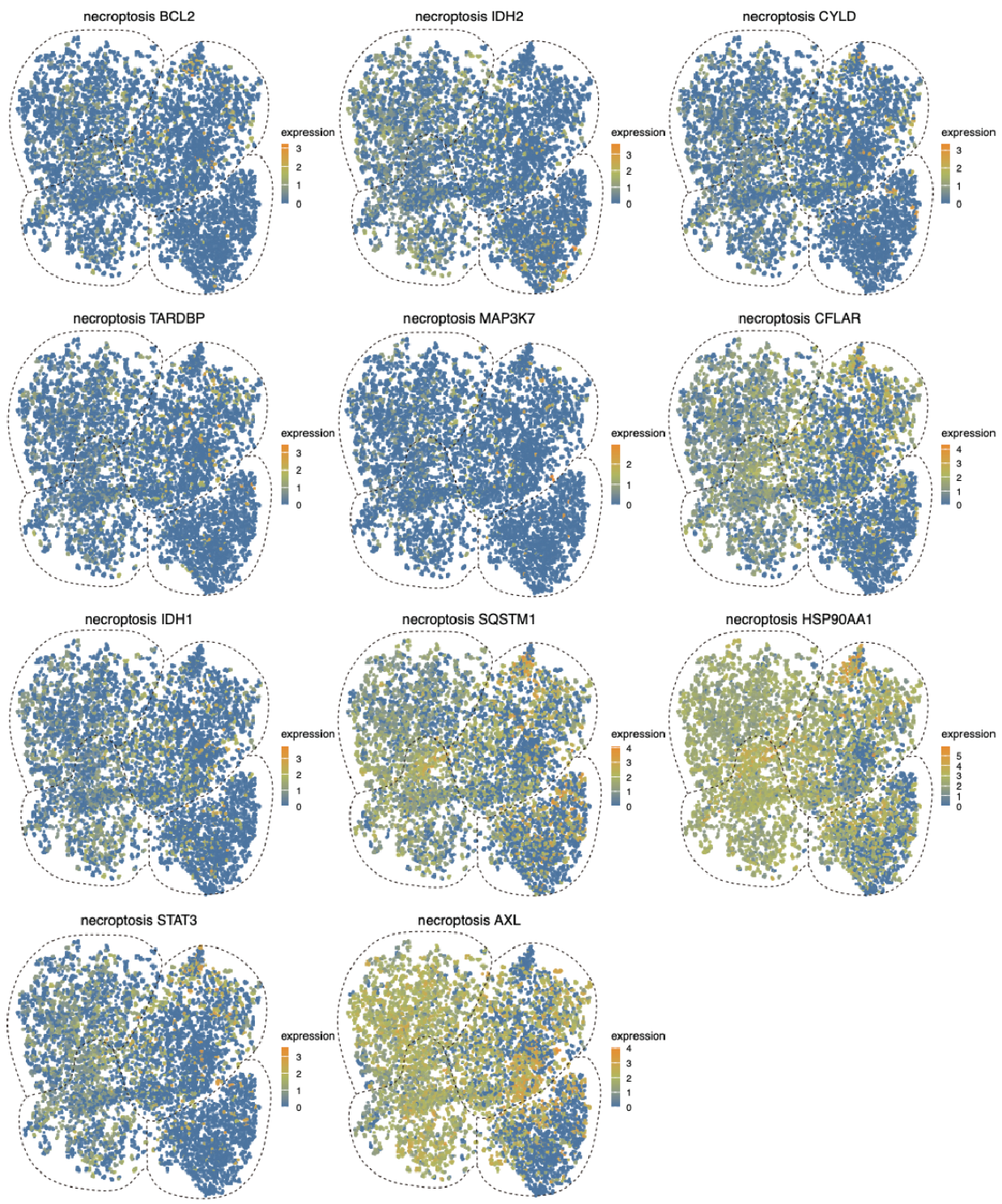

Supplement: Supplemental Material [file IANN_A_2690717_SM4886.zip › suppl_data/FigS9.png]
